# Supplementary figures and images for: ERK3/MAPK6 dictates CDC42/RAC1 activity and ARP2/3-dependent actin polymerization
Source: eLife. 2023 Apr 14;12:e85167. doi: 10.7554/eLife.85167 (PMC10191626; doi:10.7554/eLife.85167)

Figure 1

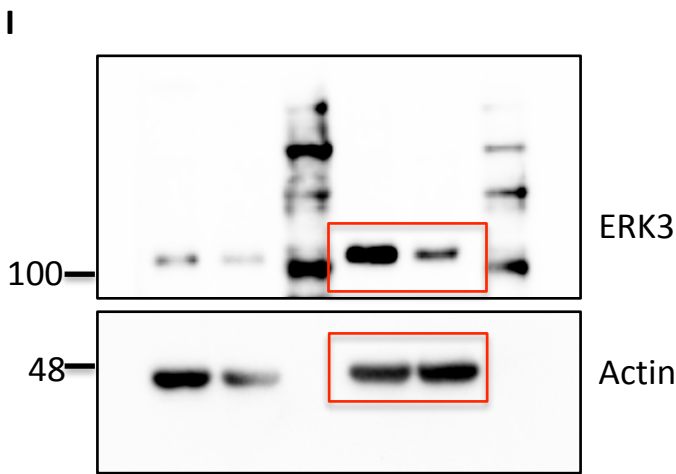

Supplement: Figure 1—source data 4. [file elife-85167-fig1-data4.zip › Figure 1-source data 4/Figure 1I-source data/Figure 1I-source data.pdf]

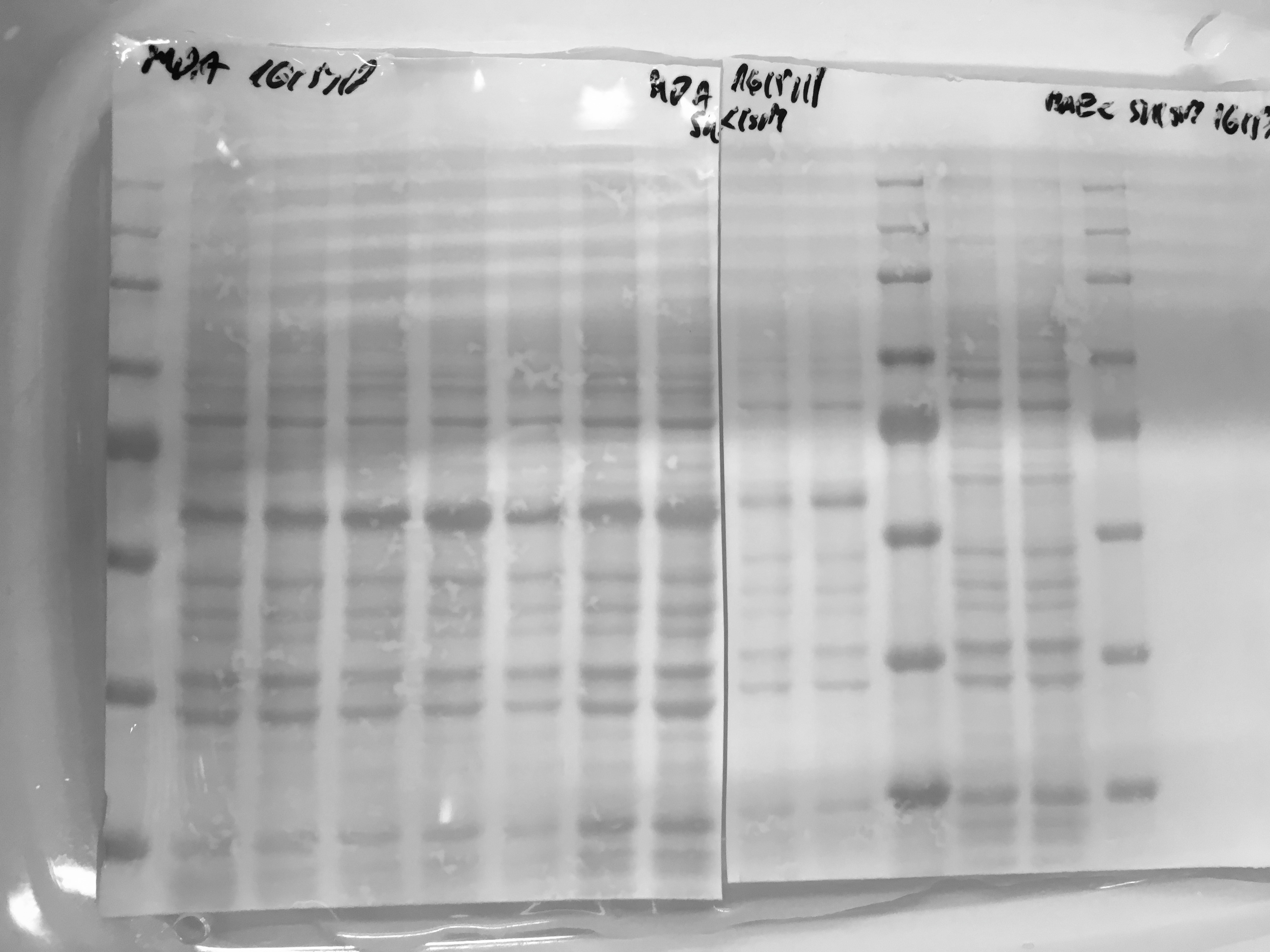

Supplement: Figure 1—source data 4. [file elife-85167-fig1-data4.zip › Figure 1-source data 4/Figure 1I-source data/originals/Ponceau S.JPG]

Figure 1-figure supplement 1

A

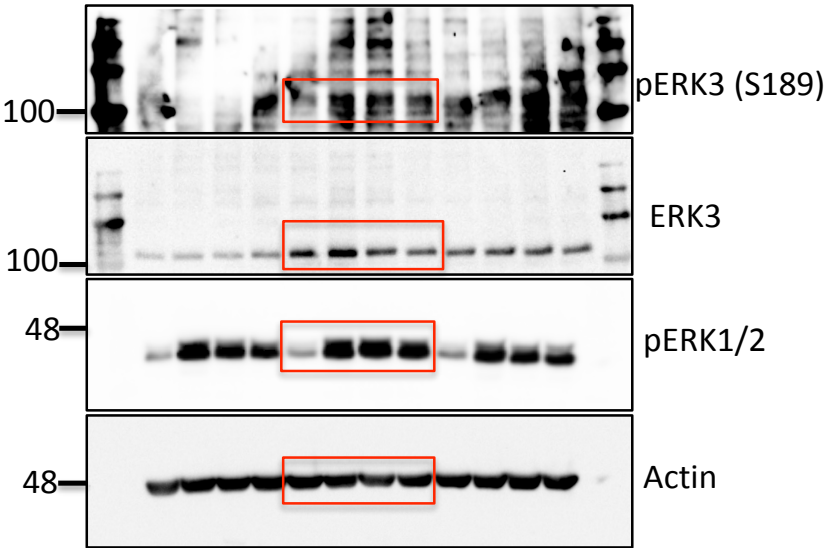

C

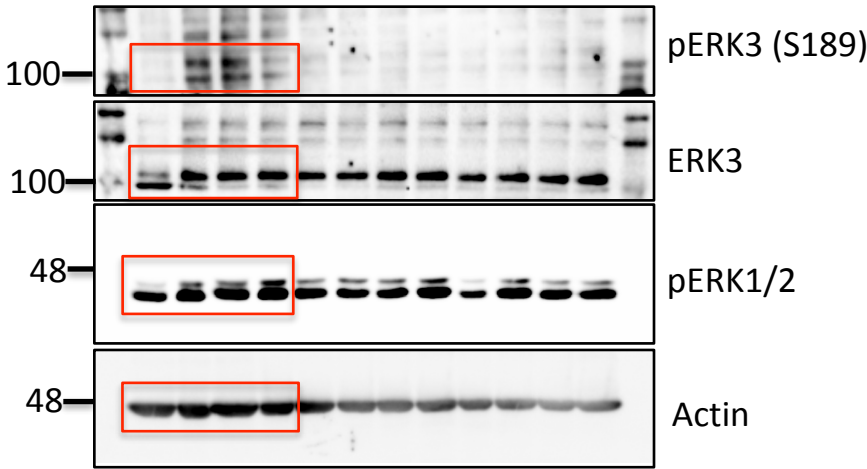

E

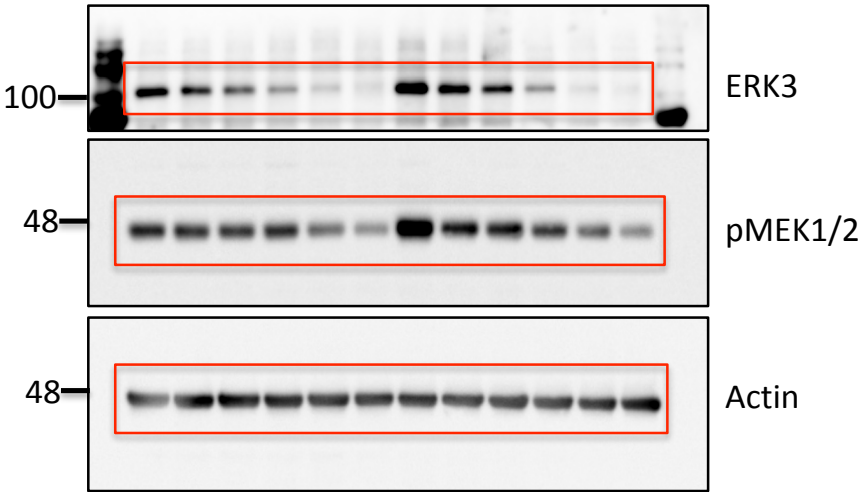

Supplement: Figure 1—figure supplement 2—source data 1. [file elife-85167-fig1-figsupp2-data1.zip › Figure 1-figure supplement 2-source data 1/Figure 1-figure supplement 2A,2C and 2E-source data.pdf]

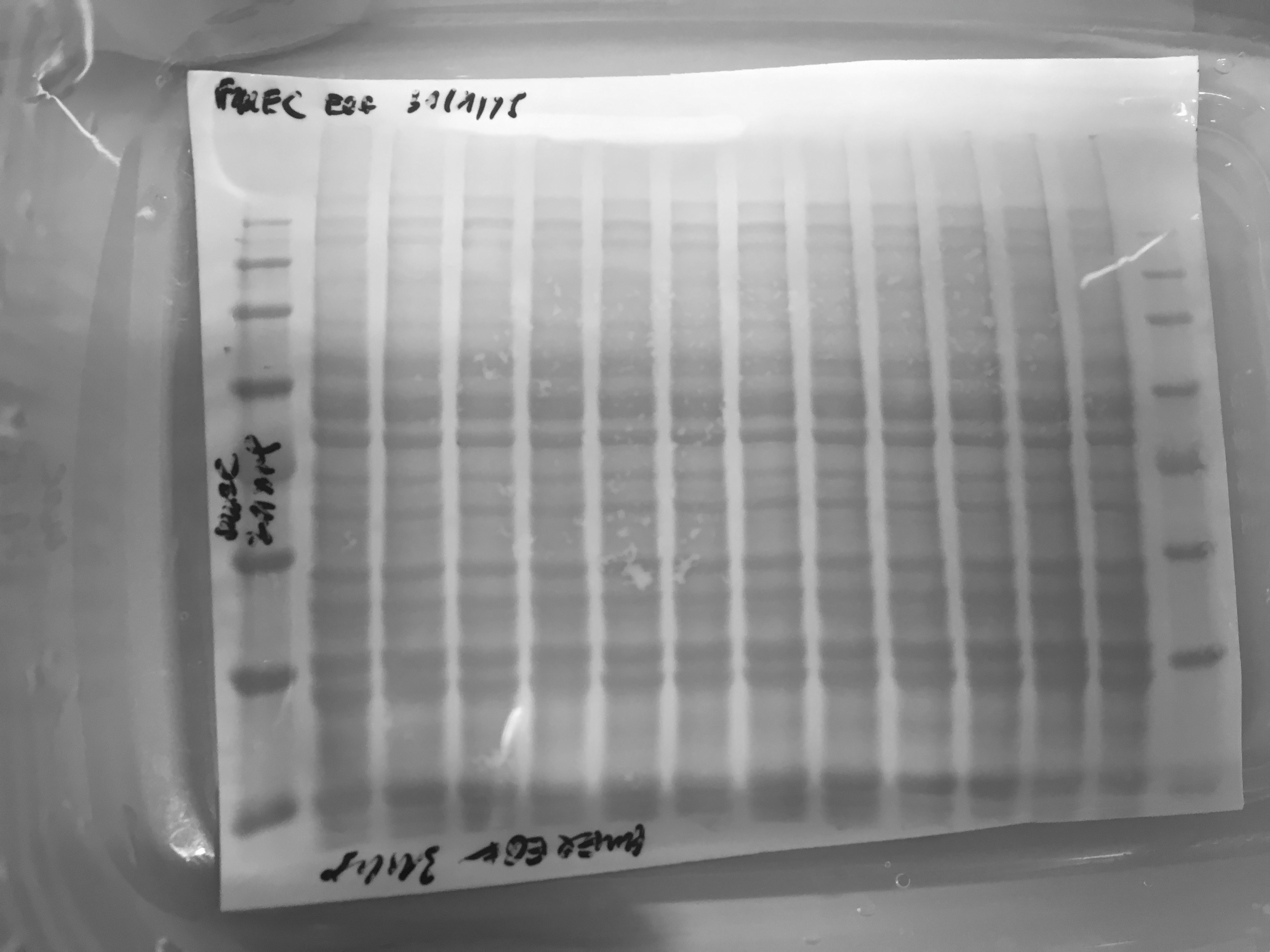

Supplement: Figure 1—figure supplement 2—source data 1. [file elife-85167-fig1-figsupp2-data1.zip › Figure 1-figure supplement 2-source data 1/Figure 1-figure supplement 2A-source data/originals/Figure 2-figure supplement 2A-source data/originals/IMG-2086.JPG]

Figure 2

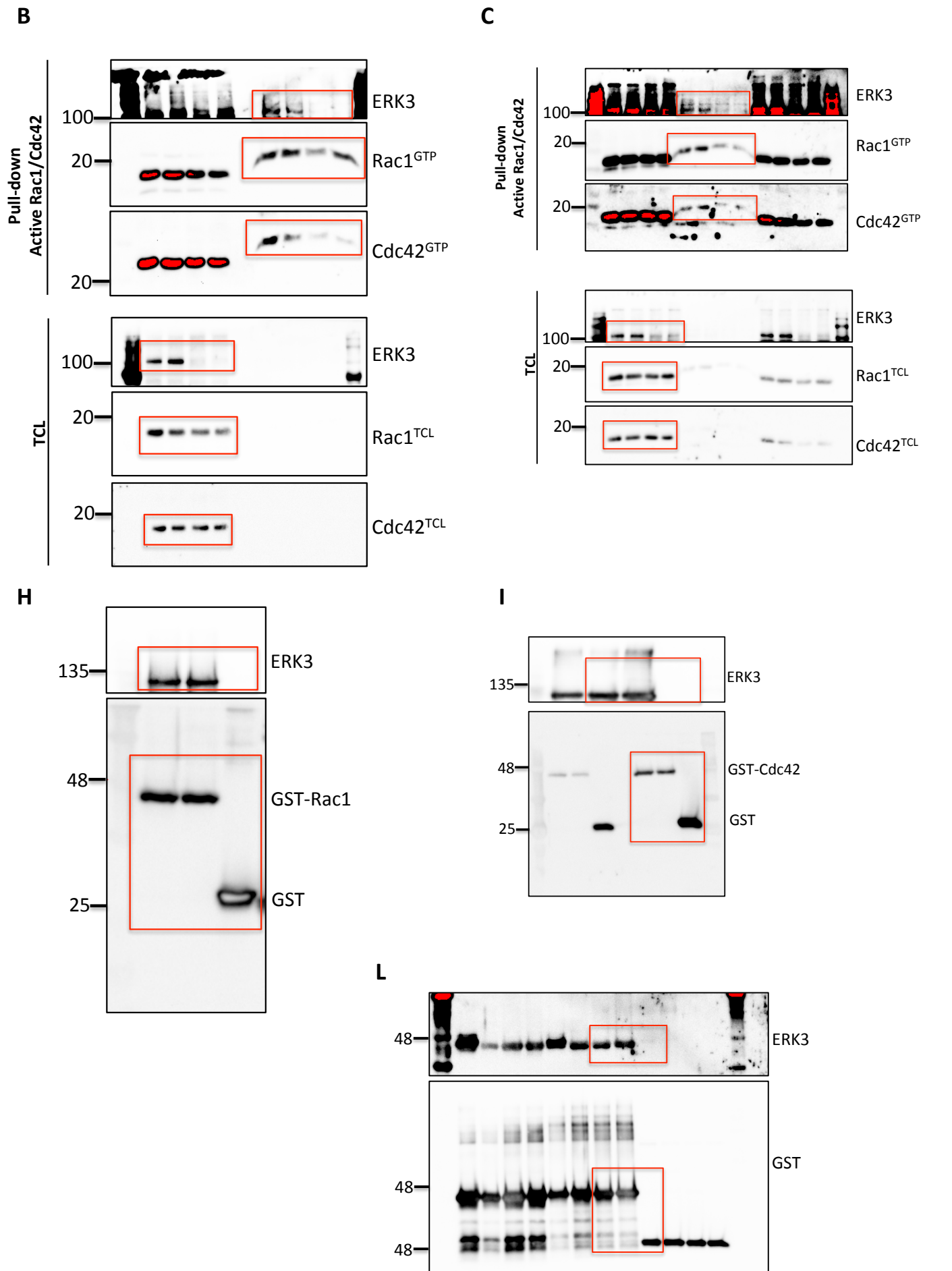

Supplement: Figure 2—source data 1. [file elife-85167-fig2-data1.zip › Figure 2-source data 1/Figure 2B,2C,2H,2I and 2L-source data.pdf]

Figure 2-figure supplement 1

A

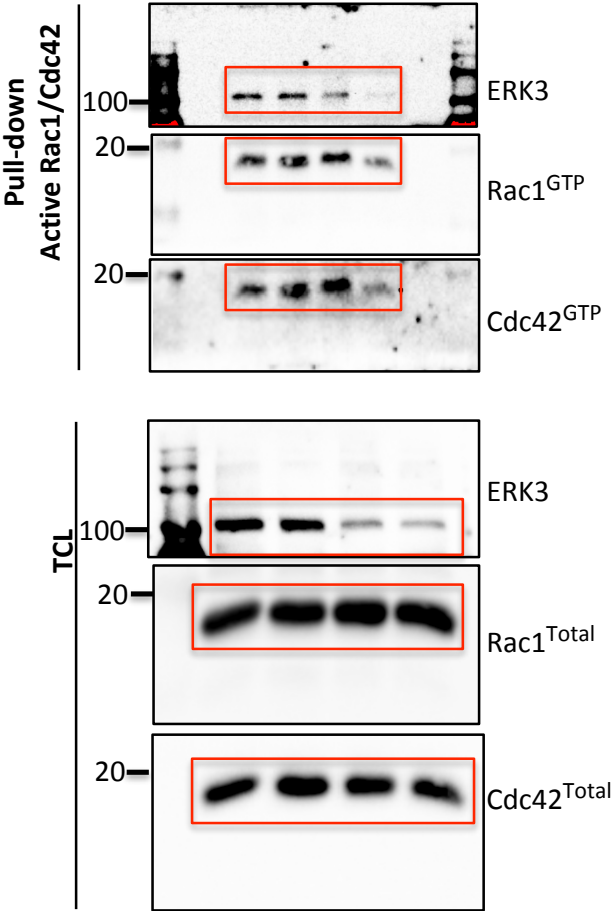

D

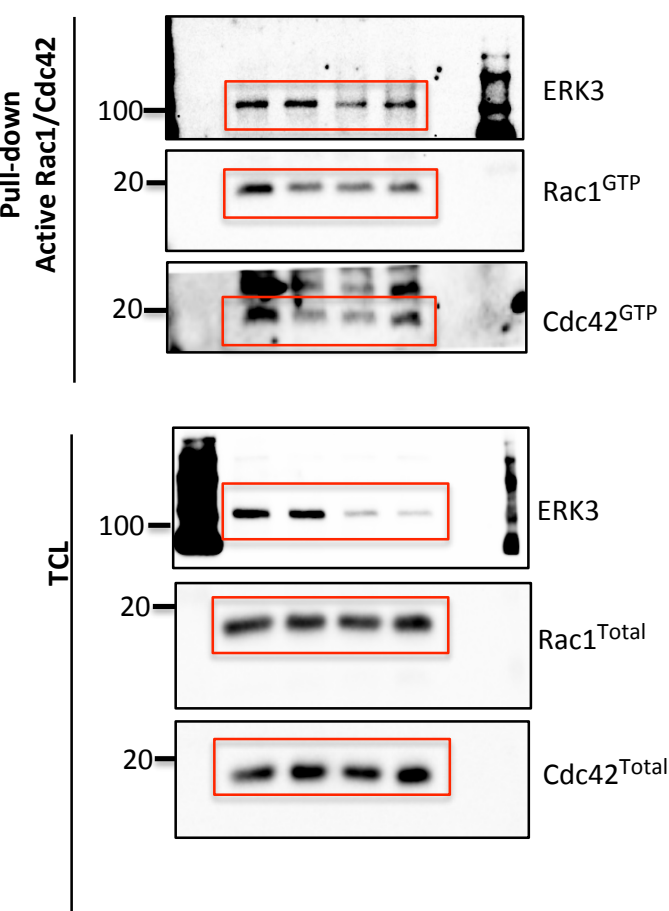

Supplement: Figure 2—figure supplement 1—source data 1. [file elife-85167-fig2-figsupp1-data1.zip › Figure 2-figure supplement 1-source data 1/Figure 2-figure supplement 1A and 1D-source data.pdf]

Figure 2-figure supplement 2

A

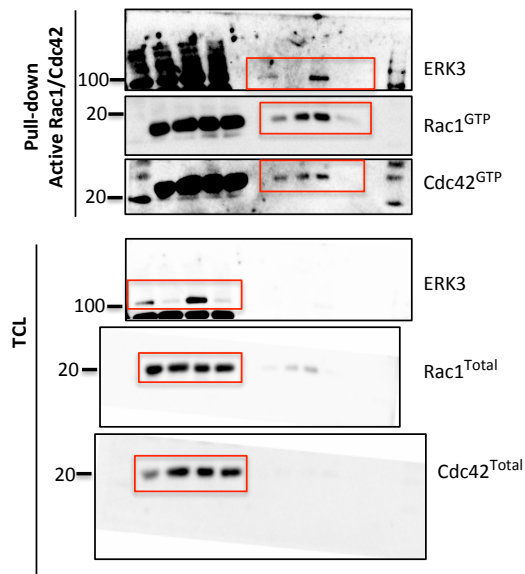

D

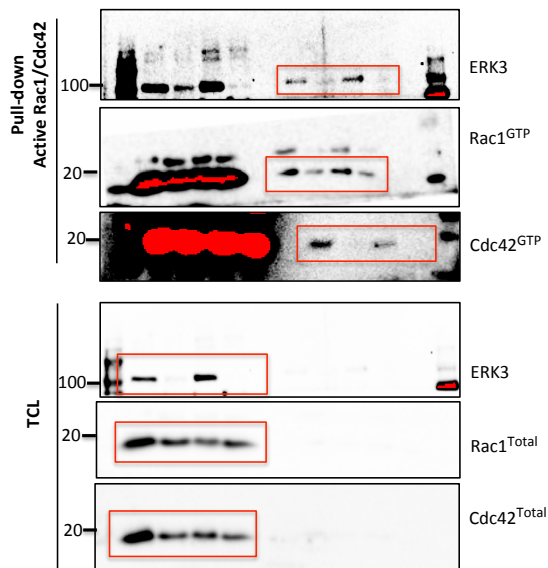

G

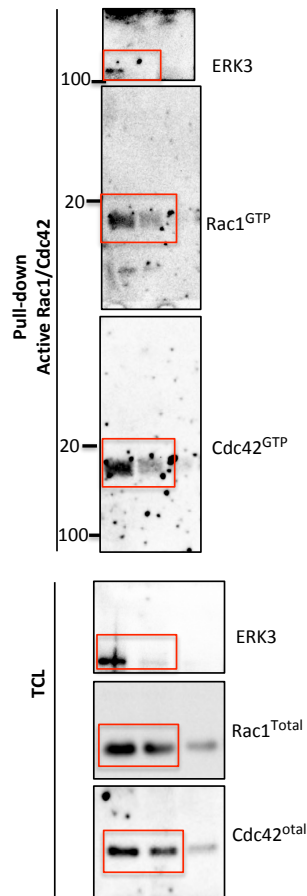

Supplement: Figure 2—figure supplement 2—source data 1. [file elife-85167-fig2-figsupp2-data1.zip › Figure 2-figure supplement 2-source data 1/Figure 2-figure supplement 2A,2D and 2G-source data.pdf]

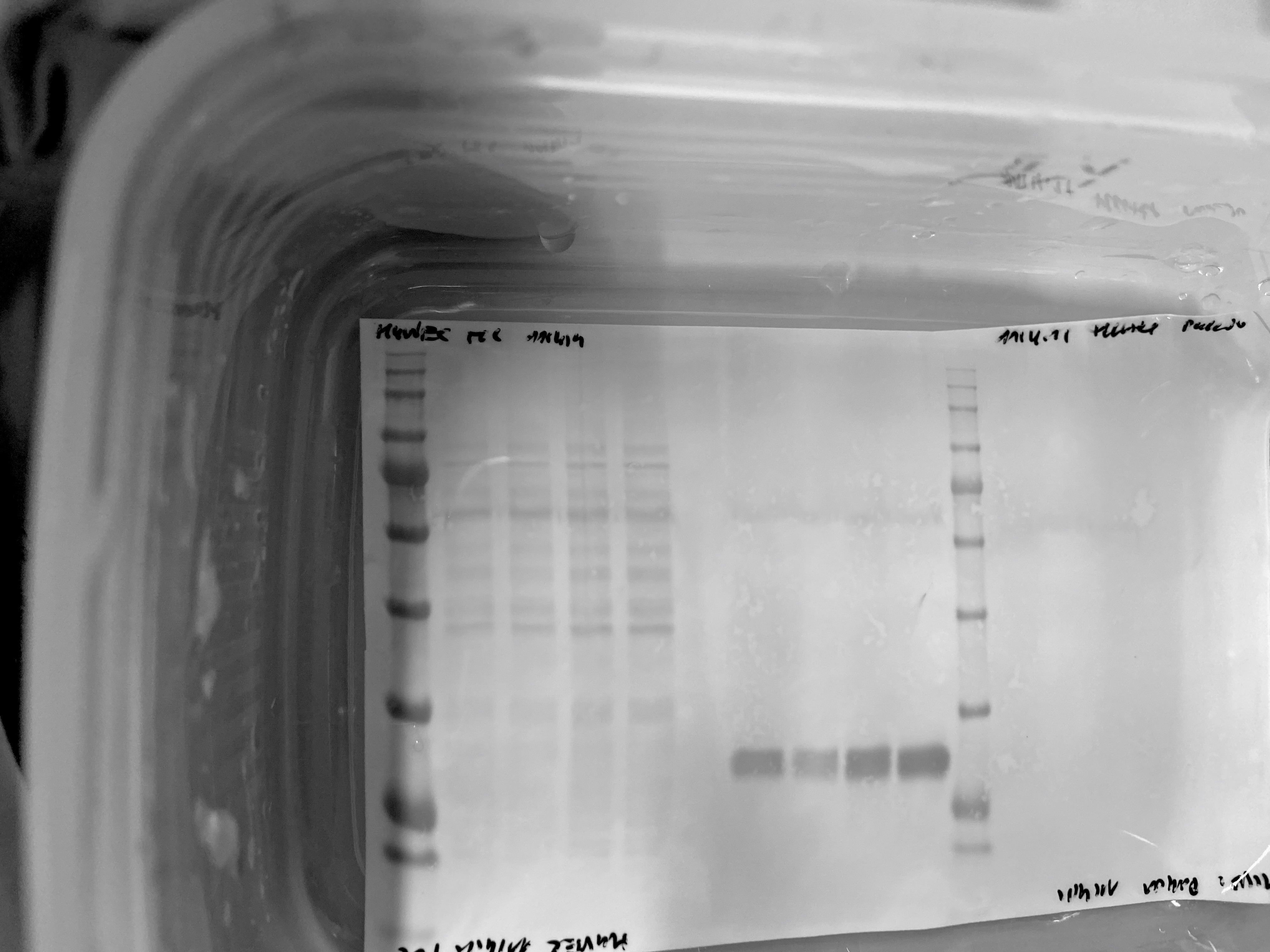

Supplement: Figure 2—figure supplement 2—source data 1. [file elife-85167-fig2-figsupp2-data1.zip › Figure 2-figure supplement 2-source data 1/Figure 2-figure supplement 2G-source data/originals/HUVEC Pak1-PBD.jpg]

Figure 2-figure supplement 3

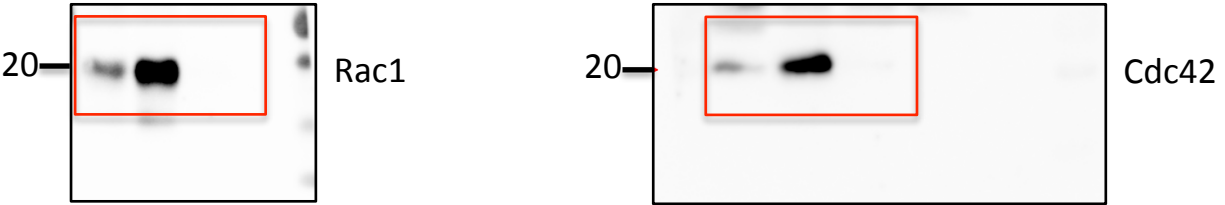

Supplement: Figure 2—figure supplement 3—source data 1. [file elife-85167-fig2-figsupp3-data1.zip › Figure 2-figure supplement 3-source data/Figure 2-figure supplement 3.pdf]

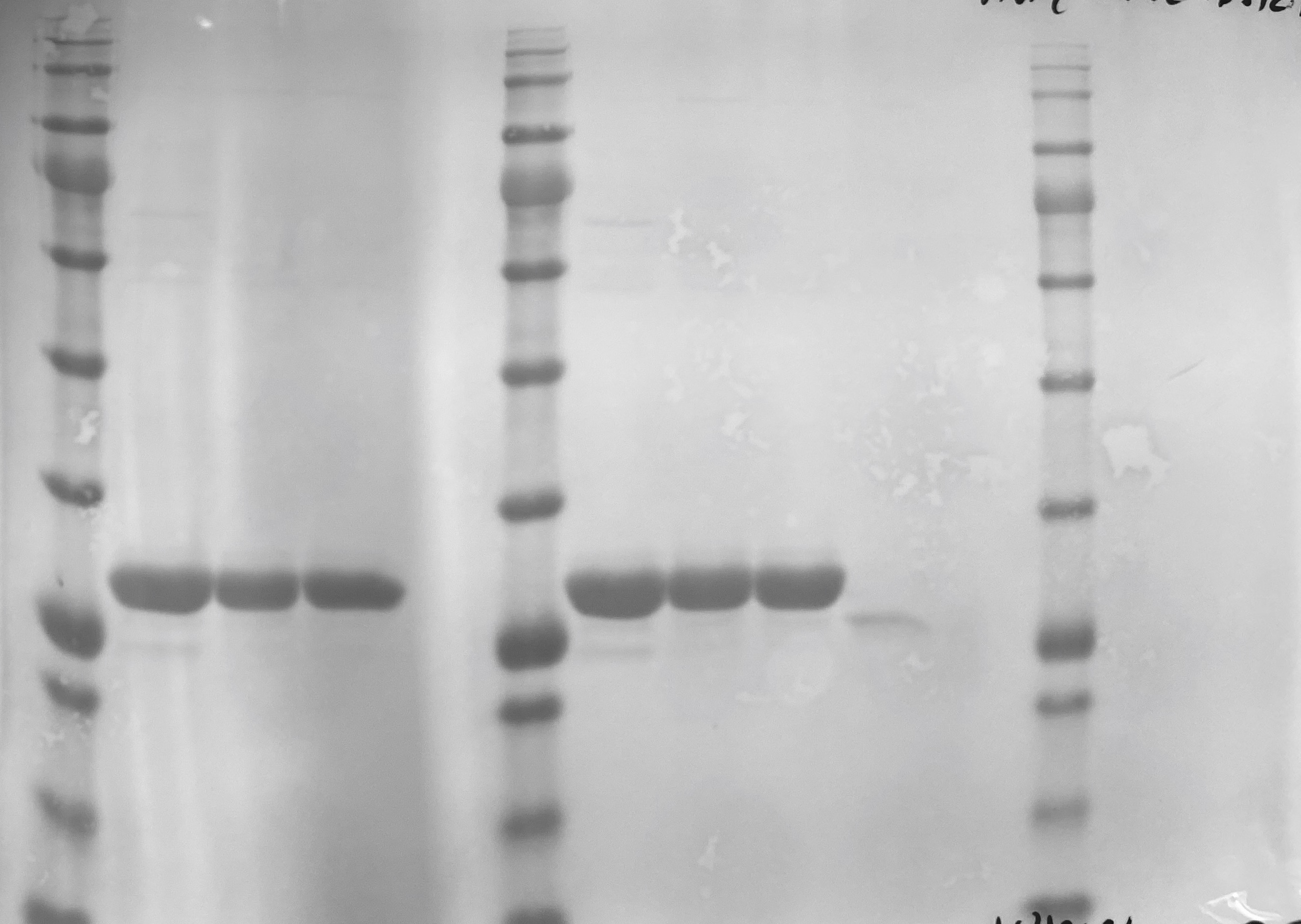

Supplement: Figure 2—figure supplement 3—source data 1. [file elife-85167-fig2-figsupp3-data1.zip › Figure 2-figure supplement 3-source data/originals/Ponceau S Pak1-PBD.jpg]

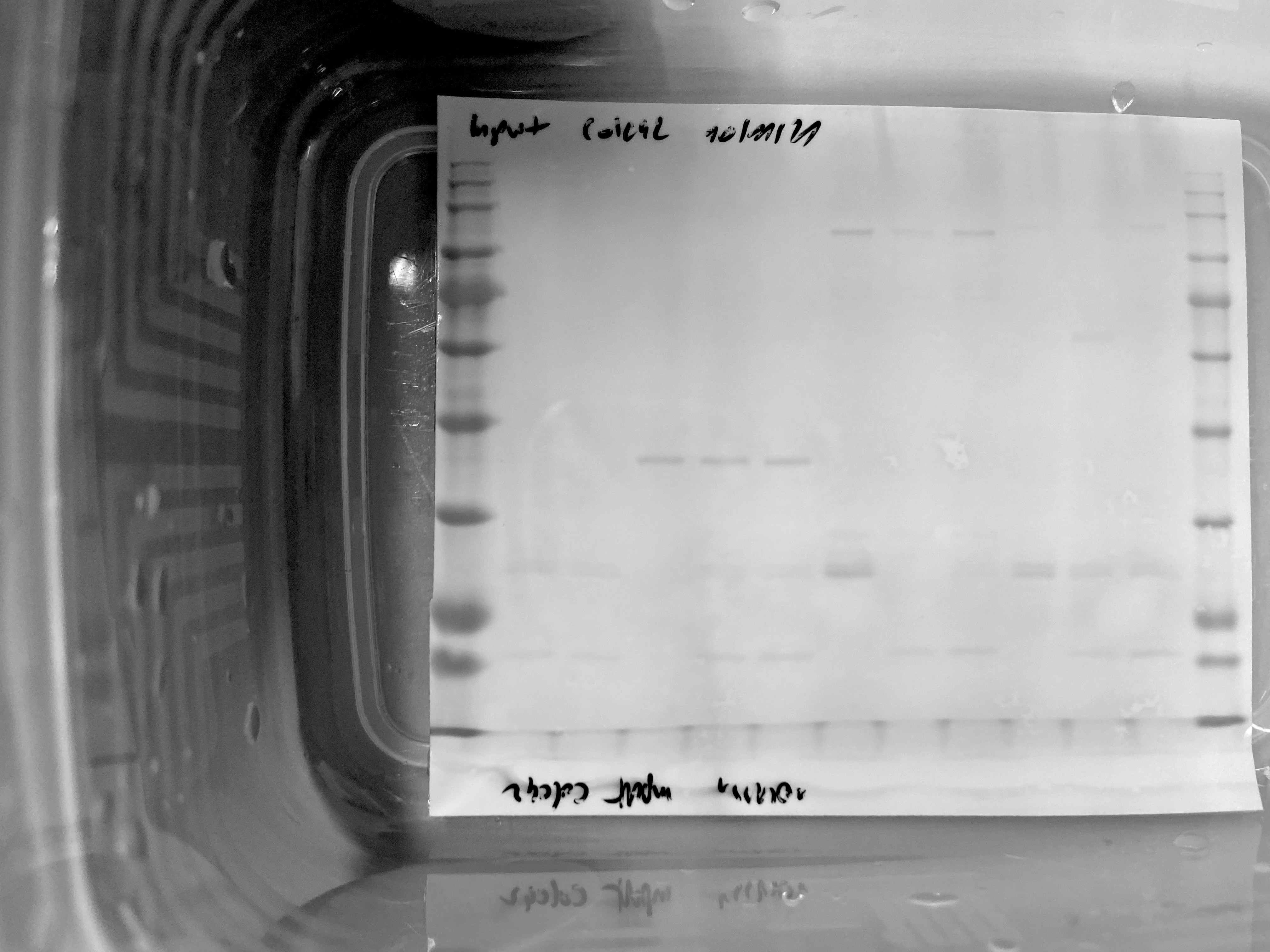

Supplement: Figure 2—figure supplement 4—source data 1. [file elife-85167-fig2-figsupp4-data1.zip › Figure 2-figure supplement 4-source data/originals/Cdc42 input.jpg]

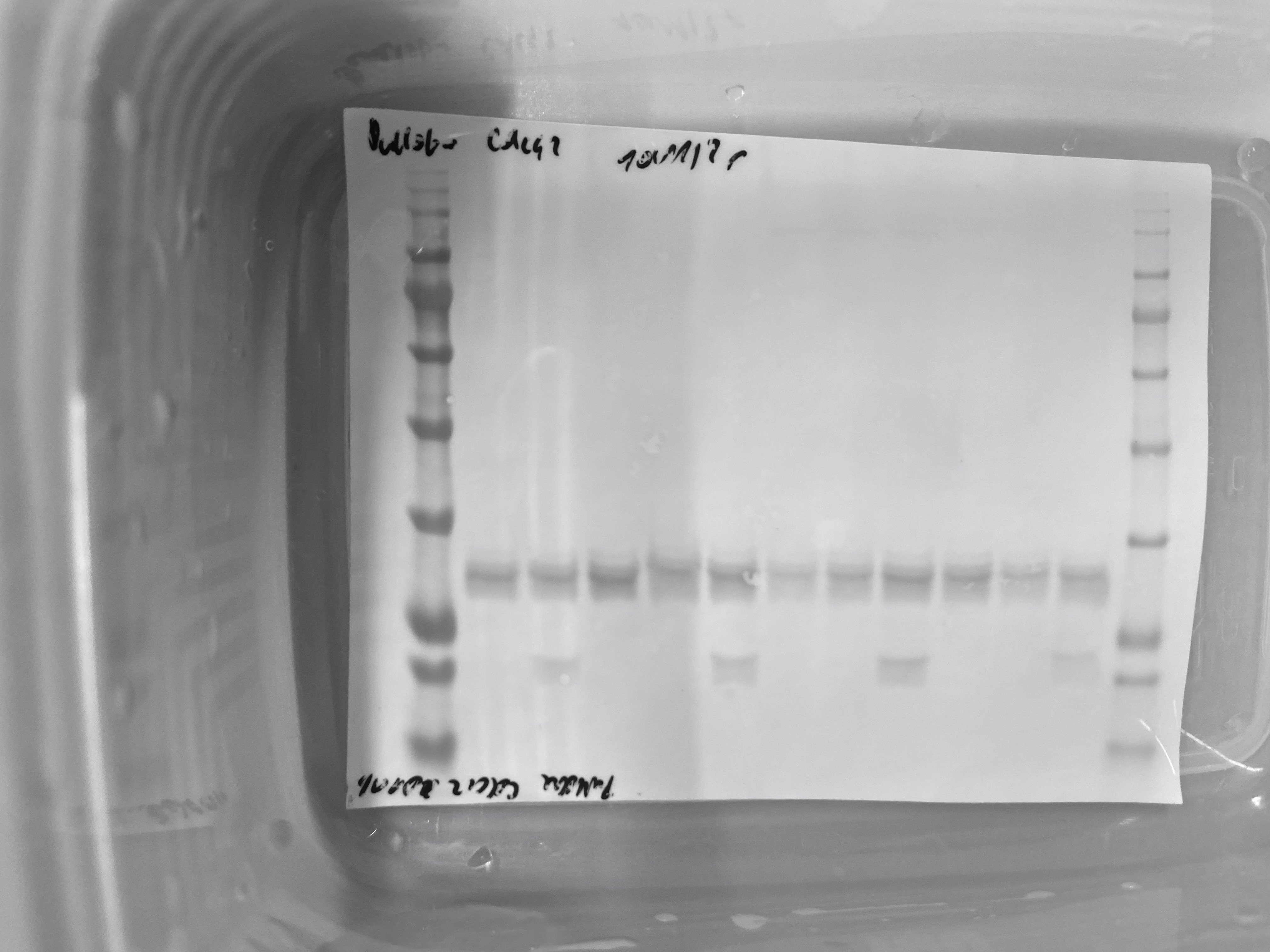

Supplement: Figure 2—figure supplement 4—source data 1. [file elife-85167-fig2-figsupp4-data1.zip › Figure 2-figure supplement 4-source data/originals/Cdc42 pulldown.jpg]

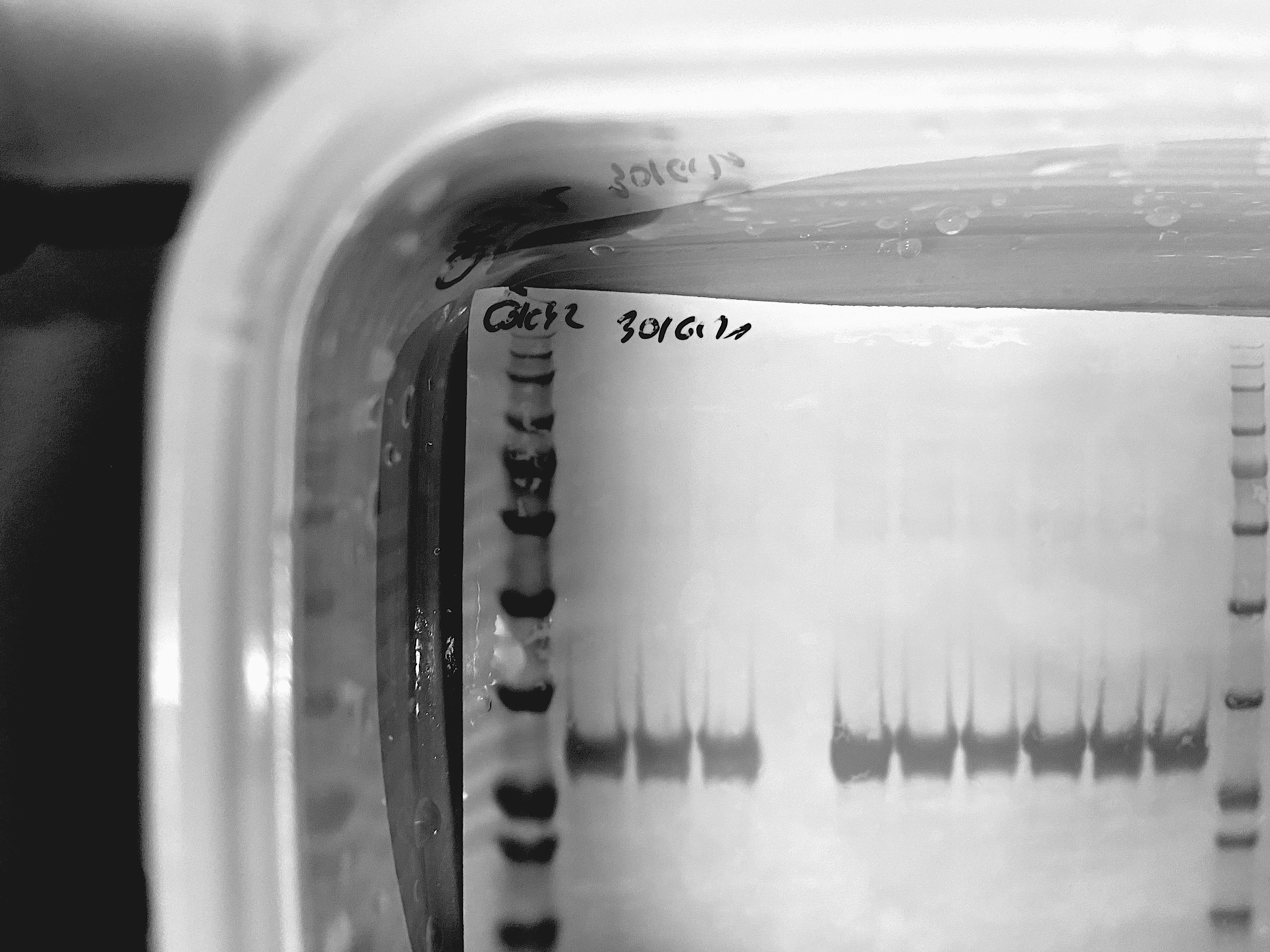

Supplement: Figure 3—source data 1. [file elife-85167-fig3-data1.zip › Figure 3-source data 1/Figure 3B-source data/originals/Pak-PBD Ponceau S.jpg]

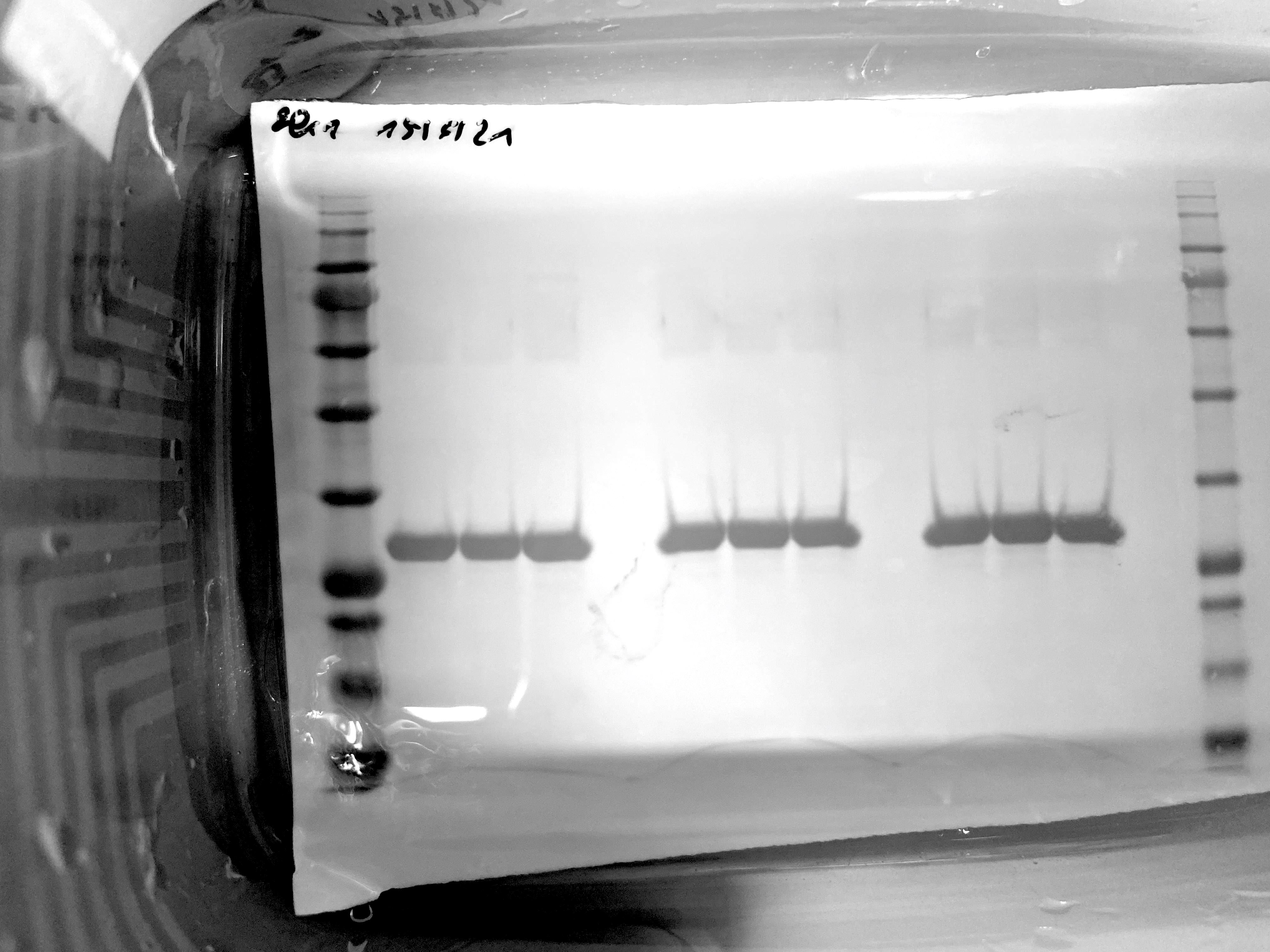

Supplement: Figure 3—source data 1. [file elife-85167-fig3-data1.zip › Figure 3-source data 1/Figure 3D-source data/originals/Pak1-PBD Ponceau S.jpg]

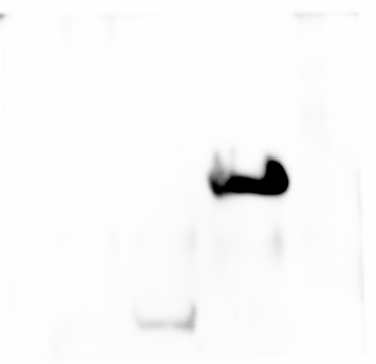

Supplement: Figure 3—figure supplement 1—source data 2. [file elife-85167-fig3-figsupp1-data2.zip › Figure 3-figure supplement 1-source data 2/Originals/MBP substrate/ERK3 kinase domain/220426 in vitro ERK3_2.tif]

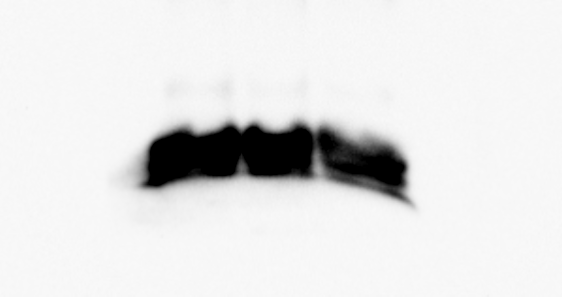

Supplement: Figure 3—figure supplement 1—source data 2. [file elife-85167-fig3-figsupp1-data2.zip › Figure 3-figure supplement 1-source data 2/Originals/MBP substrate/MBP/220404 in vtro MBP_1.tif]

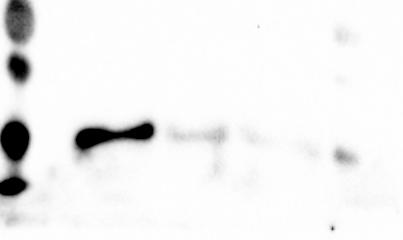

Supplement: Figure 3—figure supplement 1—source data 2. [file elife-85167-fig3-figsupp1-data2.zip › Figure 3-figure supplement 1-source data 2/Originals/MBP substrate/pSer/220331 in vitro pSER_36.tif]

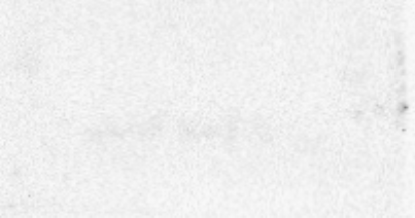

Supplement: Figure 3—figure supplement 1—source data 2. [file elife-85167-fig3-figsupp1-data2.zip › Figure 3-figure supplement 1-source data 2/Originals/MBP substrate/pThr/220401 pTHR_9 pThr.tif]

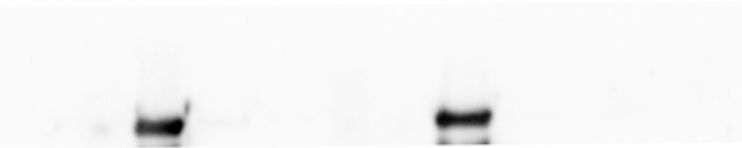

Supplement: Figure 3—figure supplement 1—source data 2. [file elife-85167-fig3-figsupp1-data2.zip › Figure 3-figure supplement 1-source data 2/Originals/MK5 substrate/ERK3/220401 in vitro ERK3_1 originals.tif]

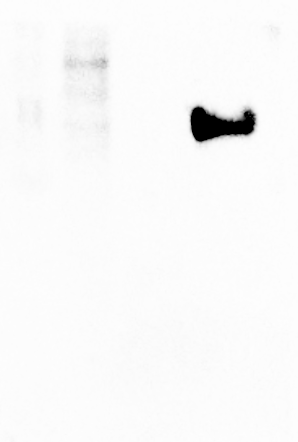

Supplement: Figure 3—figure supplement 1—source data 2. [file elife-85167-fig3-figsupp1-data2.zip › Figure 3-figure supplement 1-source data 2/Originals/MK5 substrate/ERK3 kinase domain/220401 in vitro ERK3_2 ERK3 kinase domain.tif]

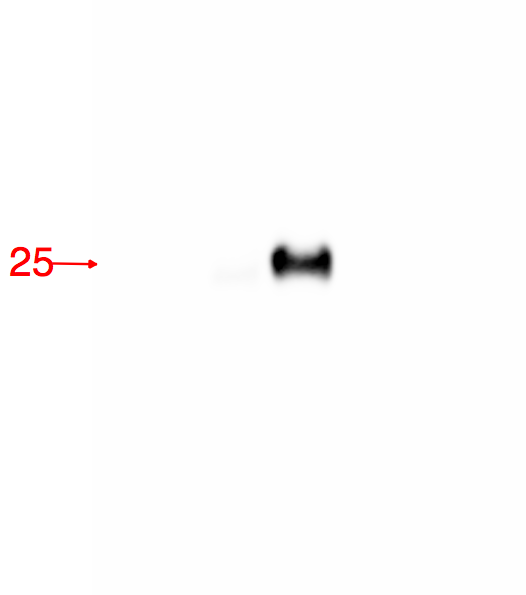

Supplement: Figure 3—figure supplement 1—source data 2. [file elife-85167-fig3-figsupp1-data2.zip › Figure 3-figure supplement 1-source data 2/Originals/MK5 substrate/GST/220404 in vitro GST_1.tif]

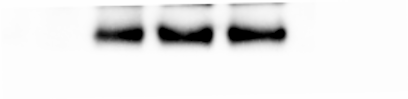

Supplement: Figure 3—figure supplement 1—source data 2. [file elife-85167-fig3-figsupp1-data2.zip › Figure 3-figure supplement 1-source data 2/Originals/MK5 substrate/MK5/220401 in vitro MK5 total_1 MK5.tif]

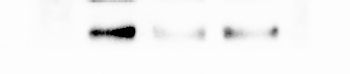

Supplement: Figure 3—figure supplement 1—source data 2. [file elife-85167-fig3-figsupp1-data2.zip › Figure 3-figure supplement 1-source data 2/Originals/MK5 substrate/pMK5 /220331 in vitro pMK5_01 pMK5.tif]

Figure 4

A

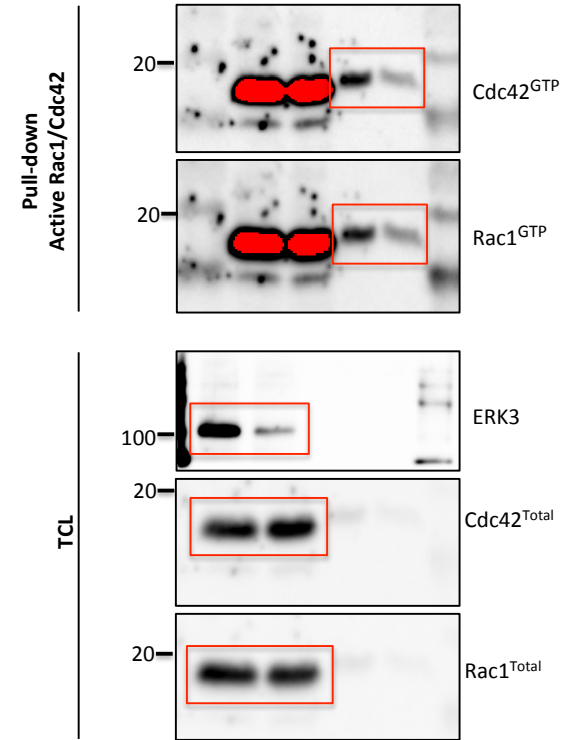

B

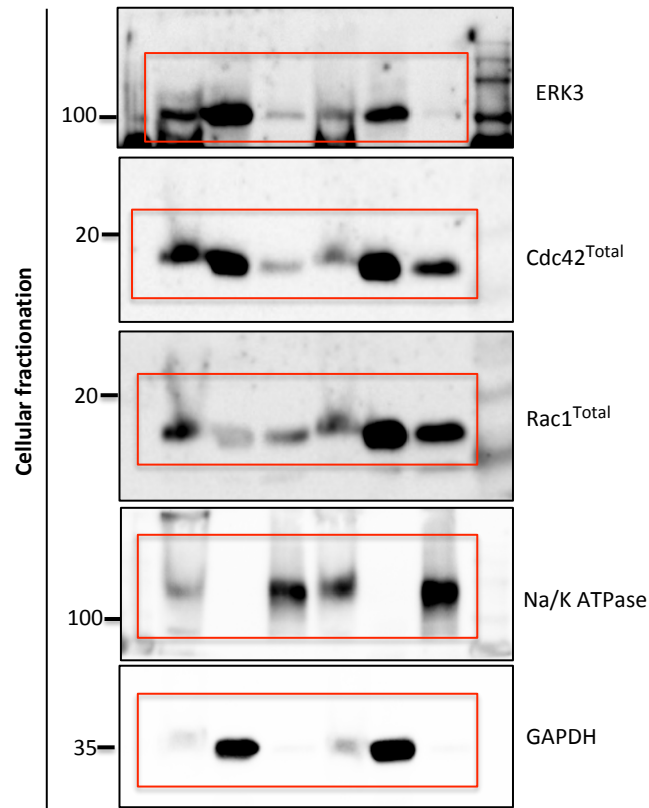

C

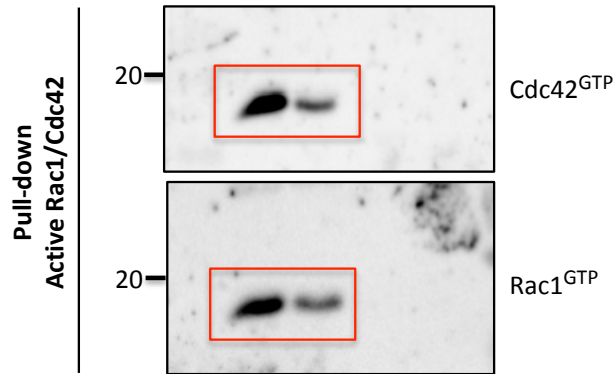

Supplement: Figure 4—source data 1. [file elife-85167-fig4-data1.zip › Figure 4-source data 1/Figure 4A,4B and 4C-source data.pdf]

Figure 5

B

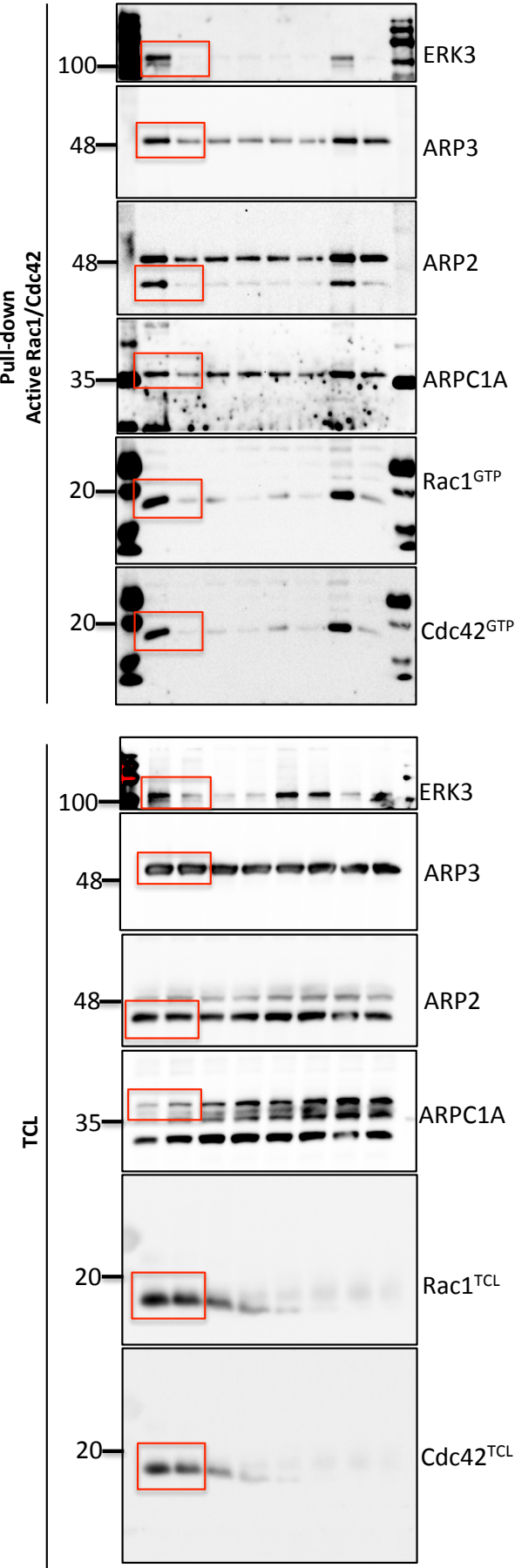

C

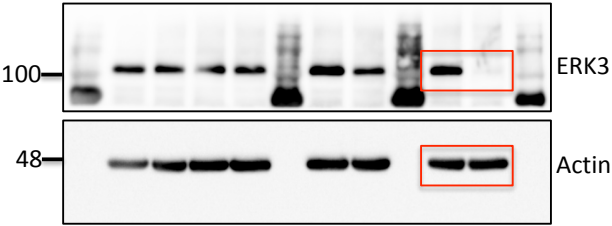

D

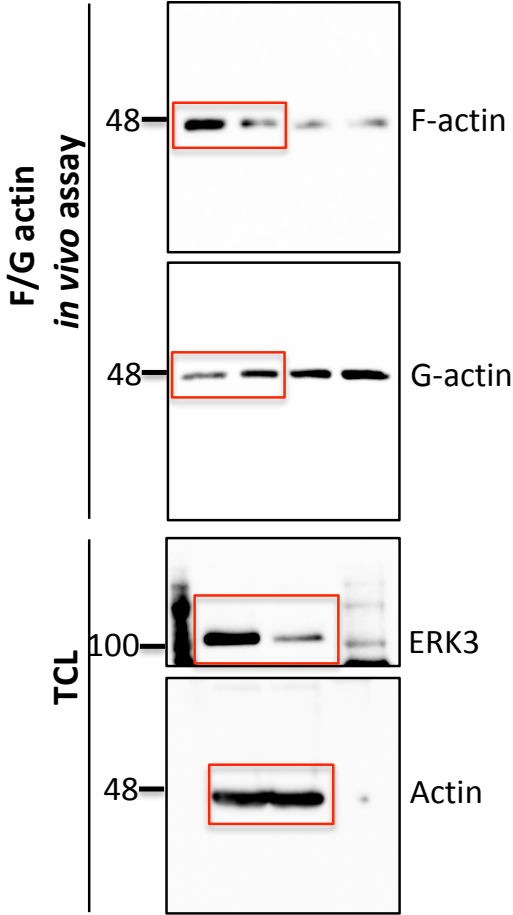

Supplement: Figure 5—source data 1. [file elife-85167-fig5-data1.zip › Figure 5-source data 1/Figure 5B,5C and 5D-source data.pdf]

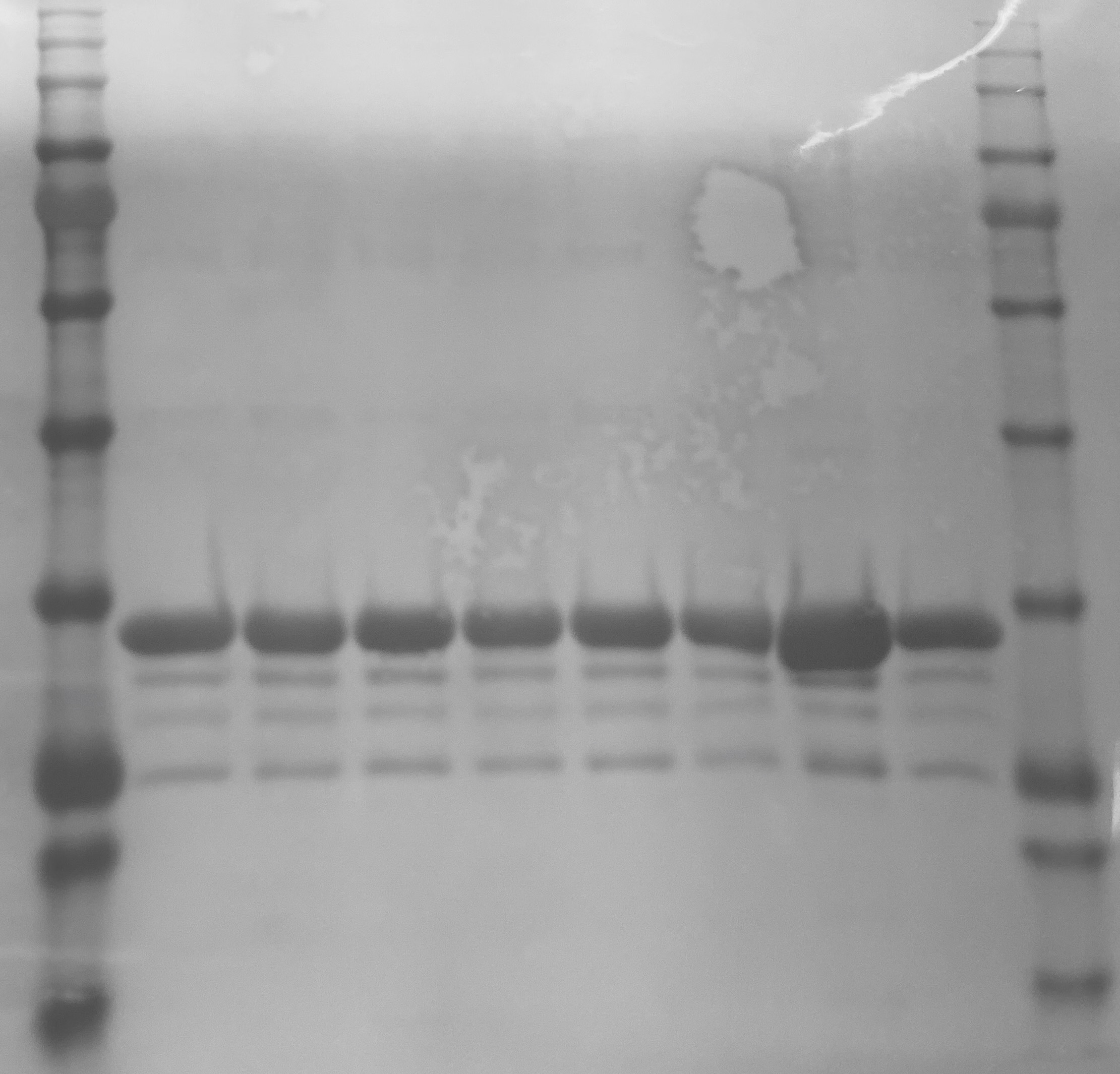

Supplement: Figure 5—source data 1. [file elife-85167-fig5-data1.zip › Figure 5-source data 1/Figure 5B-source data/originals/Ponceau S Active.jpg]

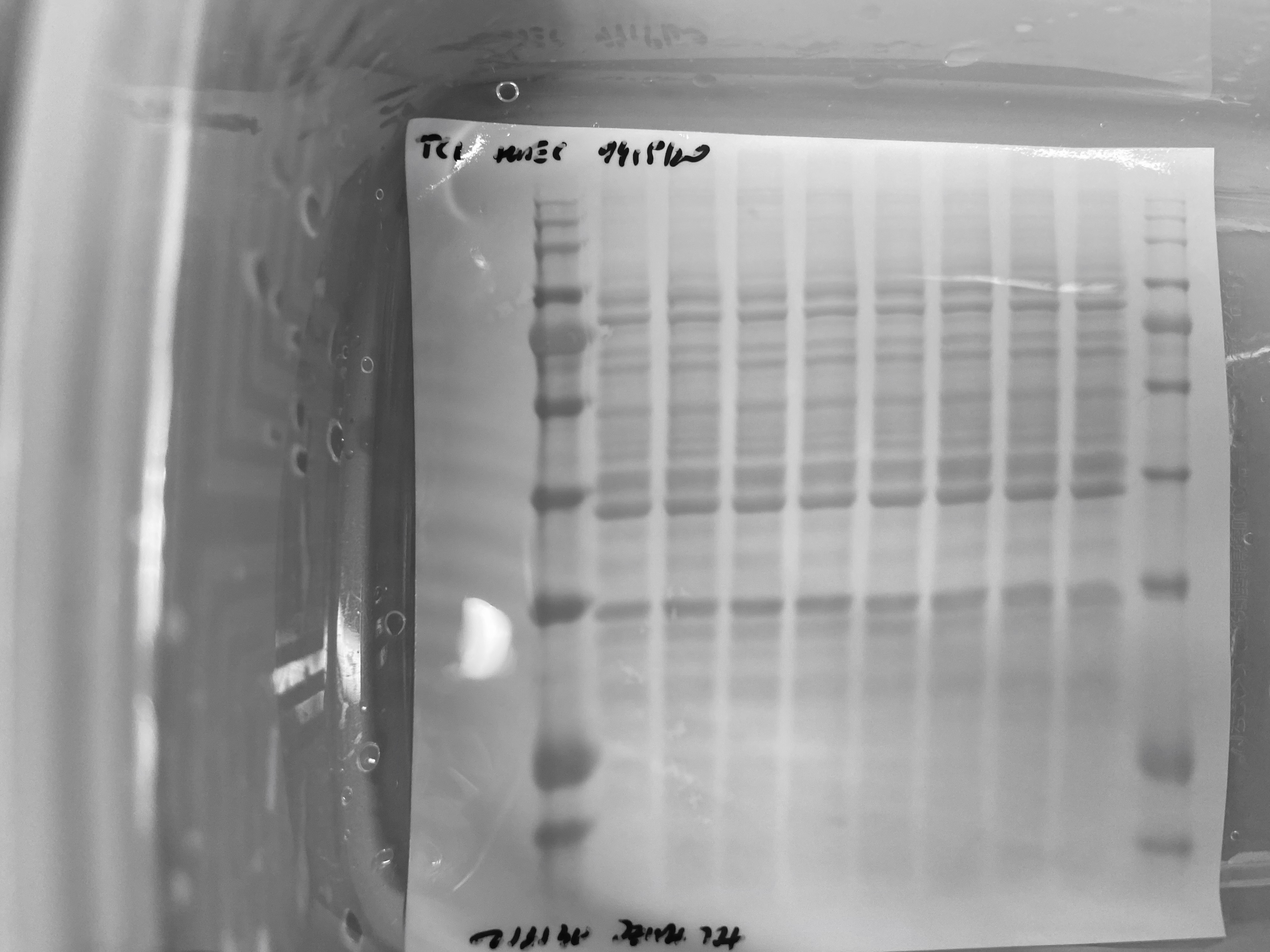

Supplement: Figure 5—source data 1. [file elife-85167-fig5-data1.zip › Figure 5-source data 1/Figure 5B-source data/originals/Ponceau S TCL.jpg]

Figure 5-figure supplement 1

B

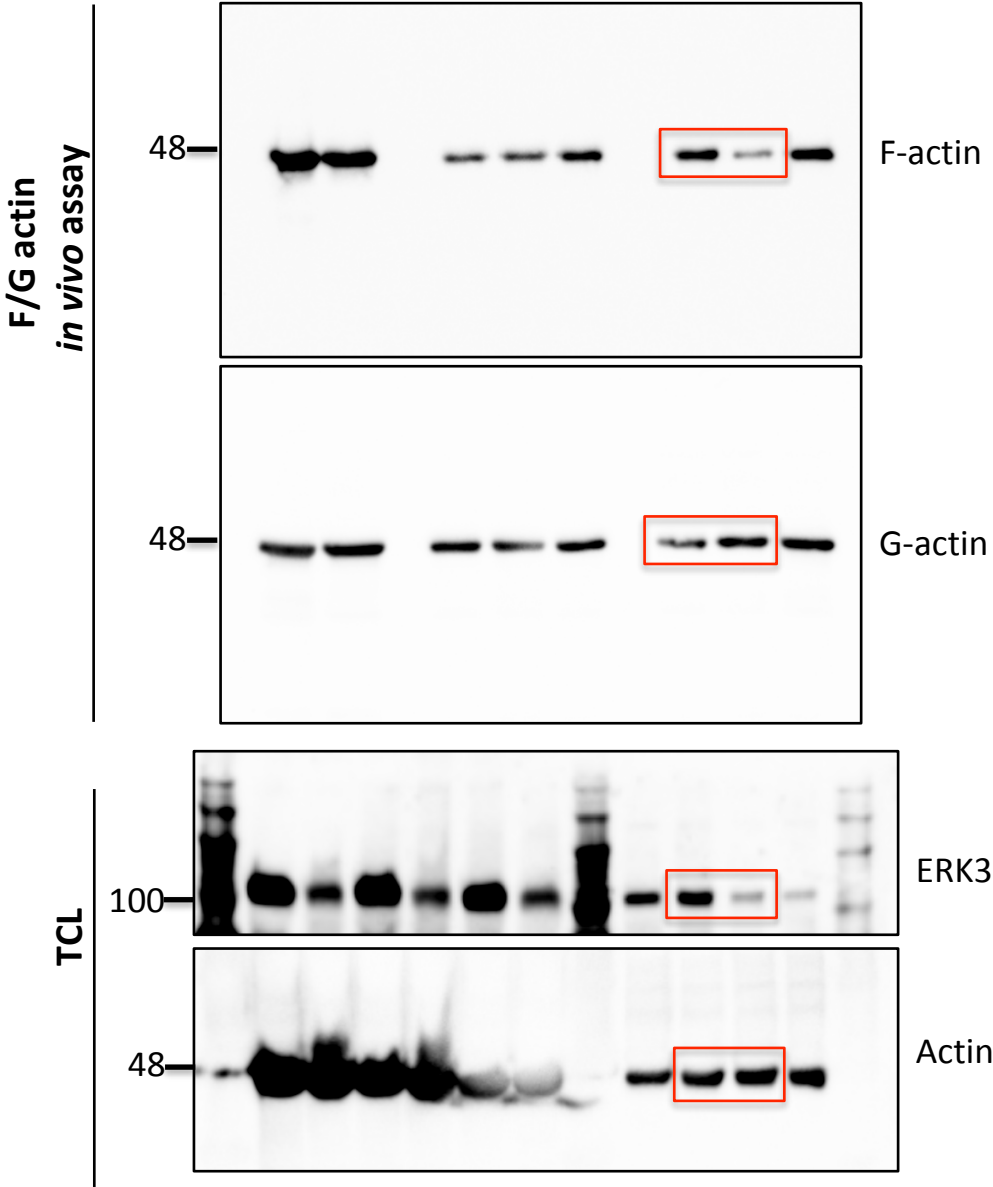

Supplement: Figure 5—figure supplement 1—source data 1. [file elife-85167-fig5-figsupp1-data1.zip › Figure 5-figure supplement 1-source data 1/Figure 5-figure supplement 1B-source data.pdf]

Figure 5-figure supplement 2

A

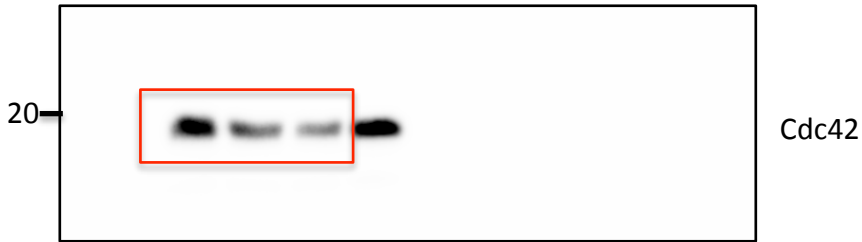

Supplement: Figure 5—figure supplement 2—source data 1. [file elife-85167-fig5-figsupp2-data1.zip › Figure 5-figure supplement 2-source data 1/Figure 5-figure supplement 2A-source data.pdf]

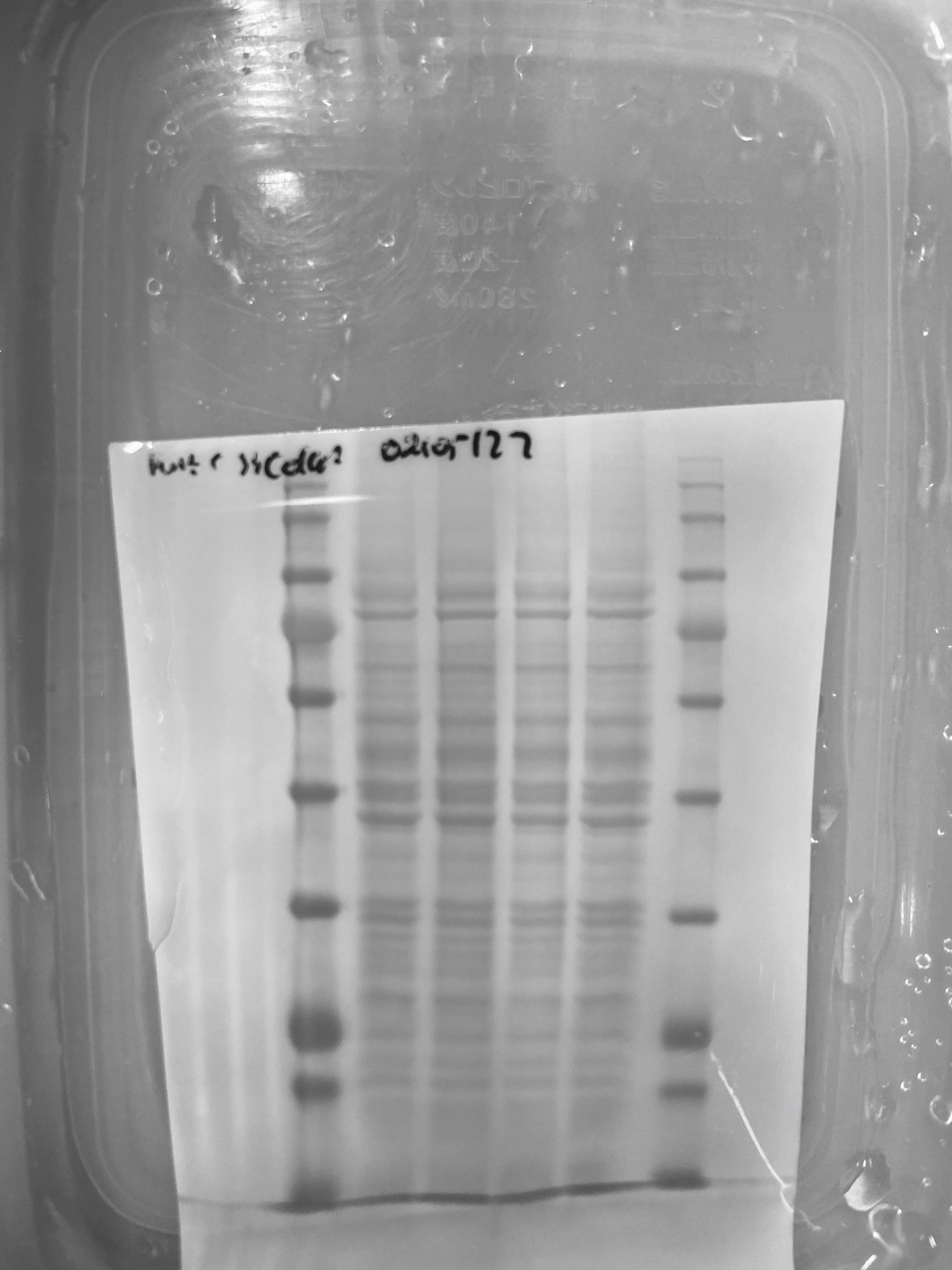

Supplement: Figure 5—figure supplement 2—source data 1. [file elife-85167-fig5-figsupp2-data1.zip › Figure 5-figure supplement 2-source data 1/originals/Ponceau s.jpg]

**Figure 6**

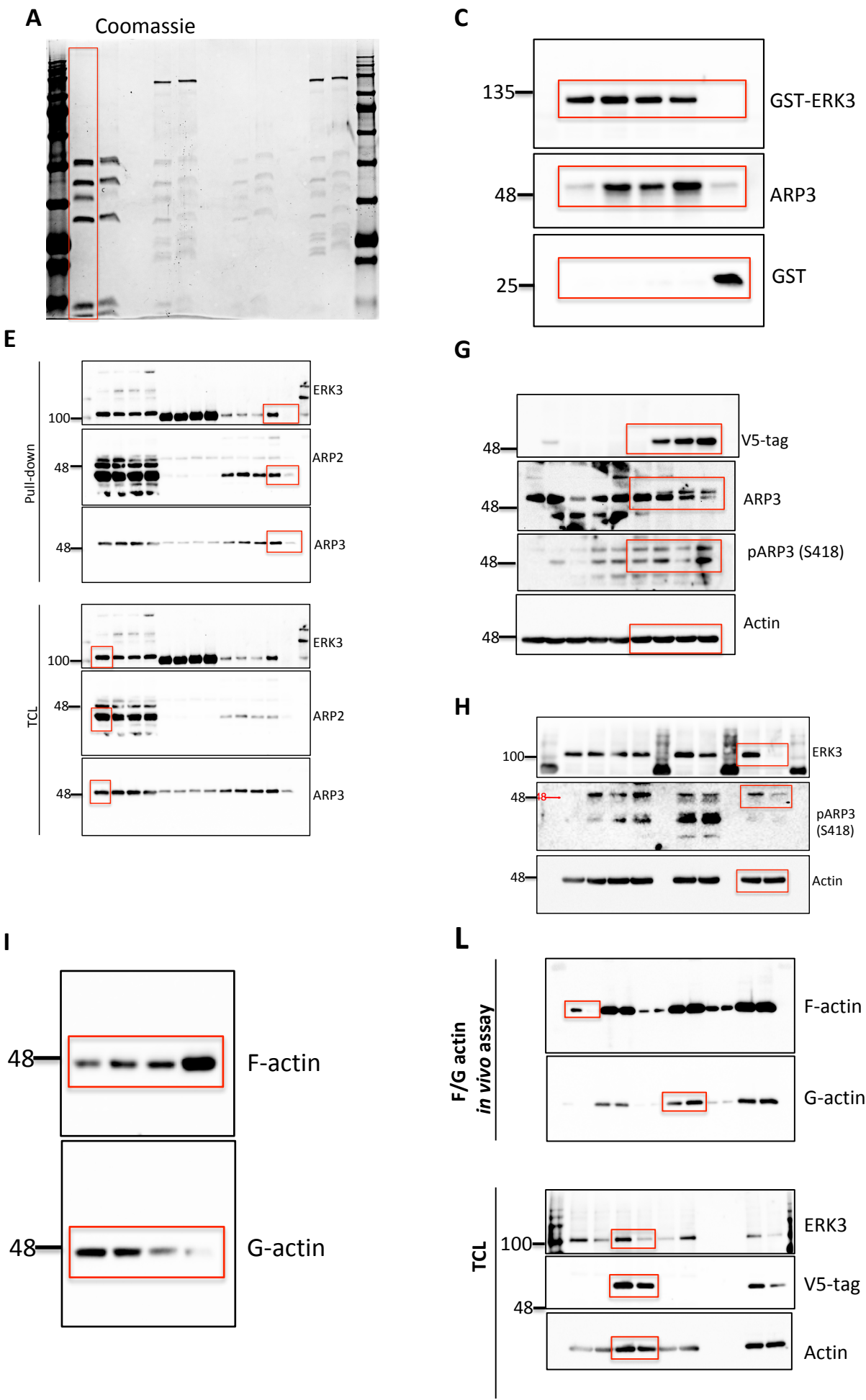

Supplement: Figure 6—source data 1. [file elife-85167-fig6-data1.zip › Figure 6-source data 1/Figure 6A, 6C, 6E, 6G, 6I and 6L-source data.pdf]

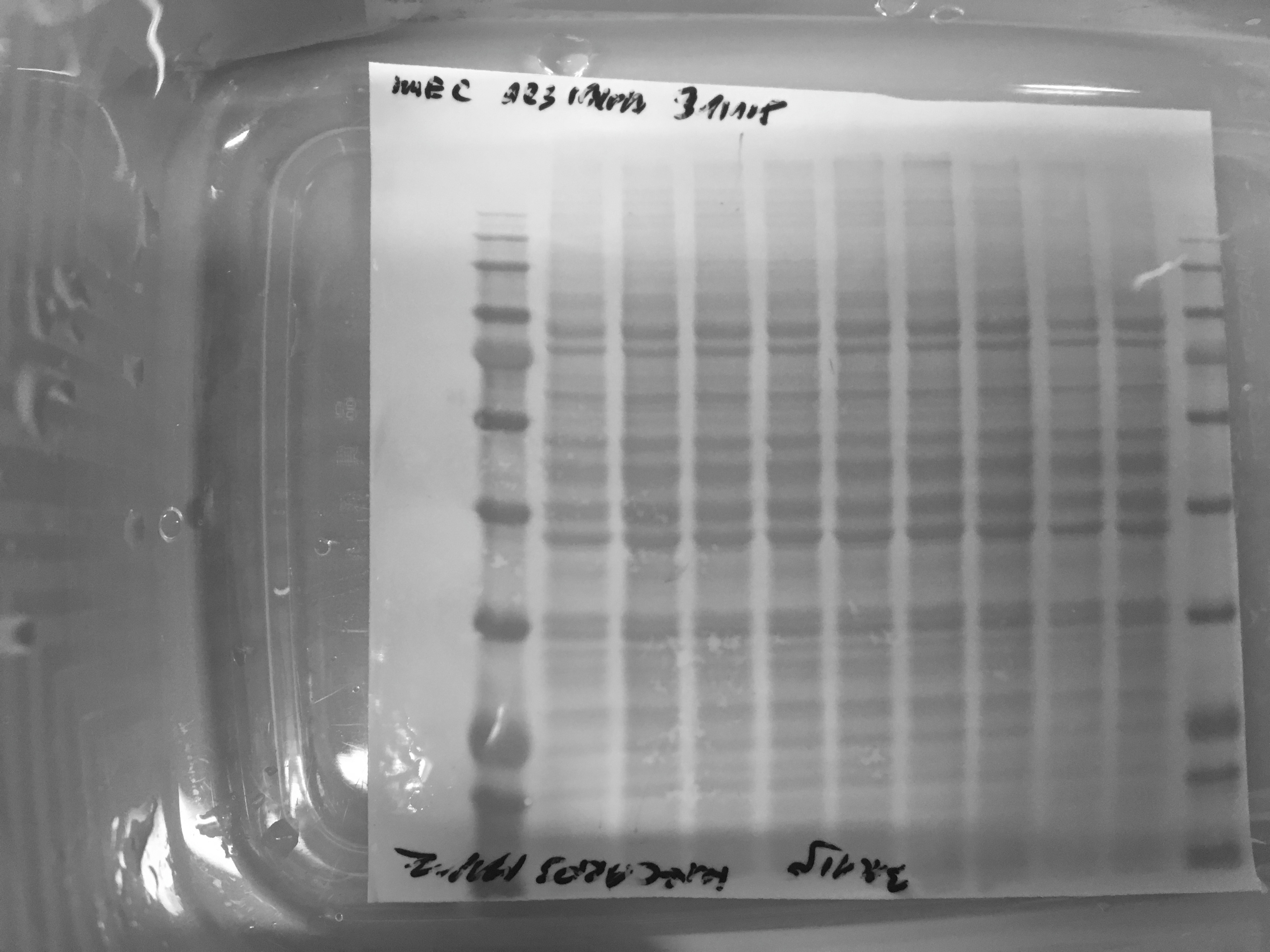

Supplement: Figure 6—source data 1. [file elife-85167-fig6-data1.zip › Figure 6-source data 1/Figure 6G-source data/originals/Ponceau S.JPG]

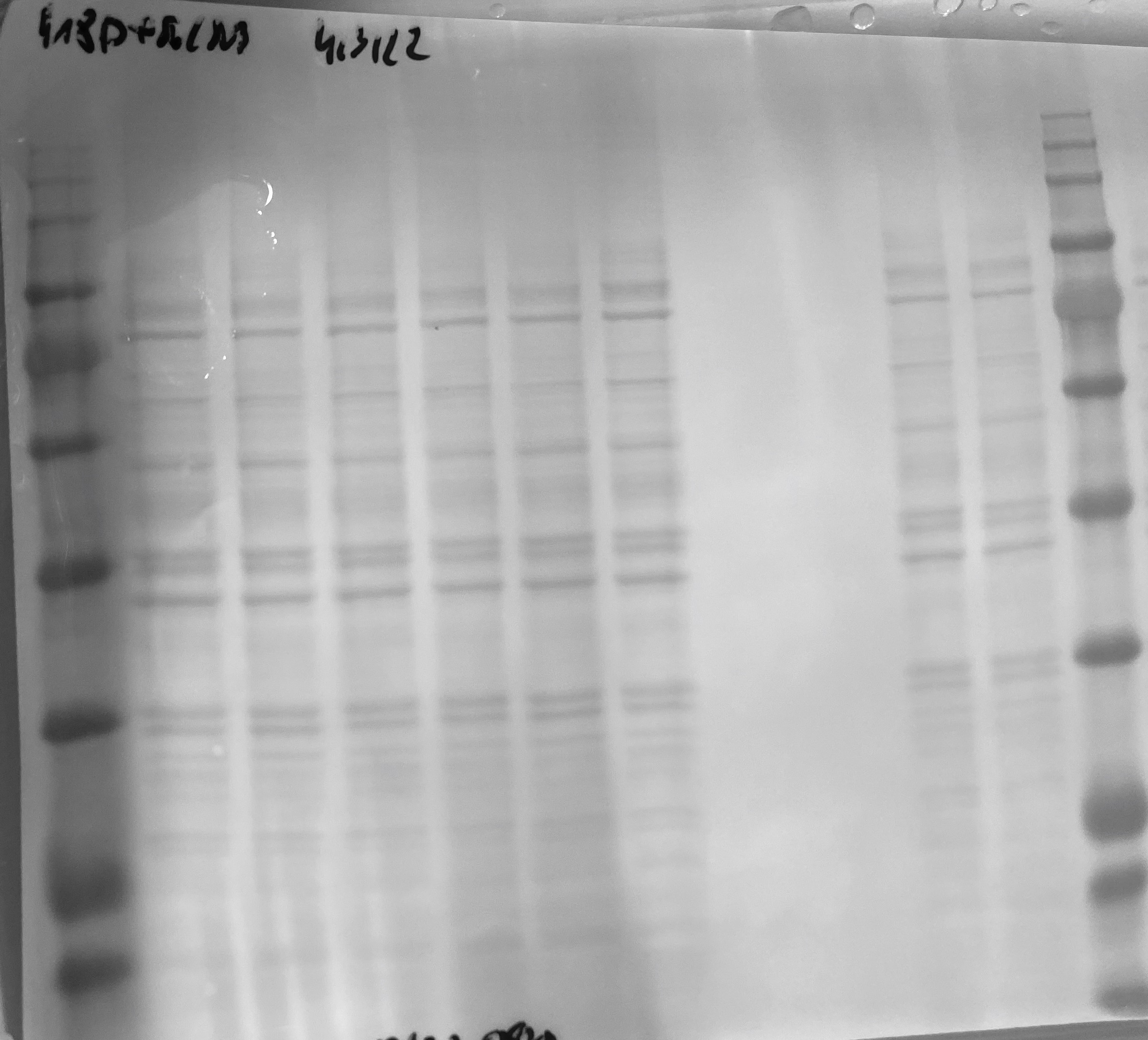

Supplement: Figure 6—source data 1. [file elife-85167-fig6-data1.zip › Figure 6-source data 1/Figure 6L-source data/Originals/TCL/HMEC EV:418D+shC:sh3.jpg]

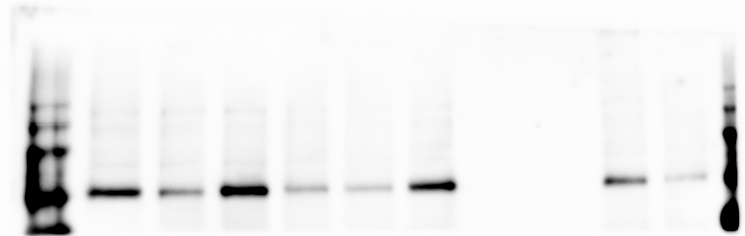

Supplement: Figure 6—source data 1. [file elife-85167-fig6-data1.zip › Figure 6-source data 1/Figure 6L-source data/Originals/TCL/HMEC S418D_shC:sh3/220307 ERK3_04 ERK3.tif]

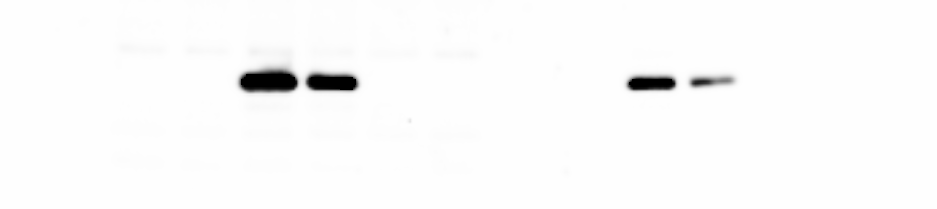

Supplement: Figure 6—source data 1. [file elife-85167-fig6-data1.zip › Figure 6-source data 1/Figure 6L-source data/Originals/TCL/HMEC S418D_shC:sh3/220307 V5_17 V5 longer.tif]

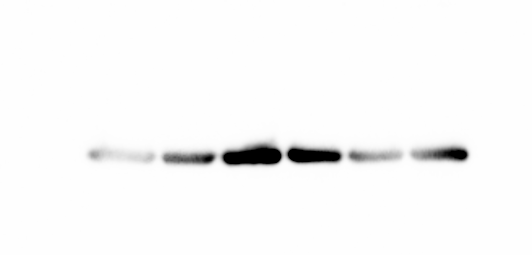

Supplement: Figure 6—source data 1. [file elife-85167-fig6-data1.zip › Figure 6-source data 1/Figure 6L-source data/Originals/TCL/HMEC S418D_shC:sh3/220524 ACTIN HRP_04 Actin HRP.tif]

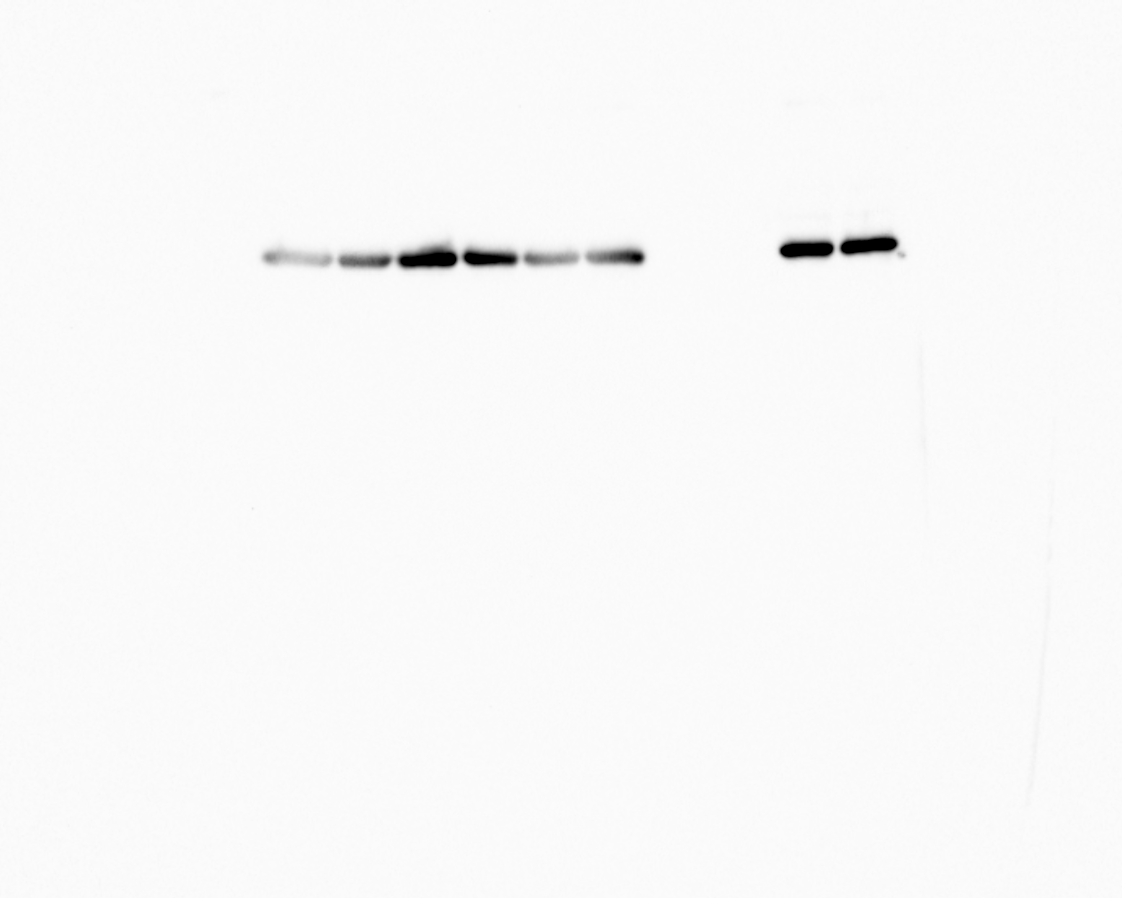

Supplement: Figure 6—source data 1. [file elife-85167-fig6-data1.zip › Figure 6-source data 1/Figure 6L-source data/Originals/TCL/HMEC S418D_shC:sh3/220524 ACTIN HRP_04 Actin originals.tif]

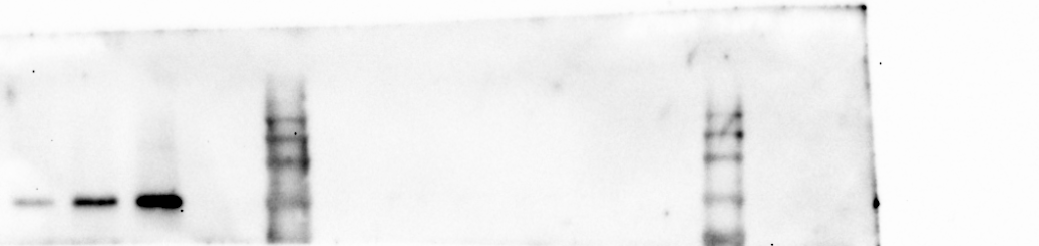

Supplement: Figure 6—figure supplement 1—source data 2. [file elife-85167-fig6-figsupp1-data2.zip › Figure 6-figure supplement 1-source data 2/Originals/ERK3/220505 ERK3 reprobed_24 ERK3.tif]

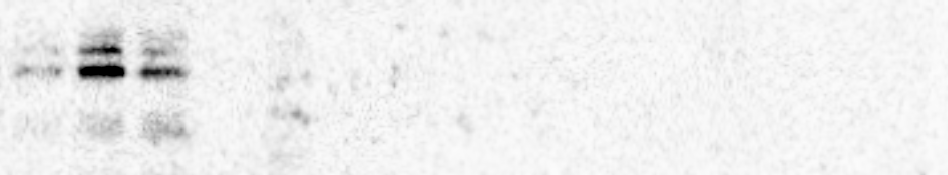

Supplement: Figure 6—figure supplement 1—source data 2. [file elife-85167-fig6-figsupp1-data2.zip › Figure 6-figure supplement 1-source data 2/Originals/pARP3 (S418)/220920 ARP3 S418 2_4.tif]

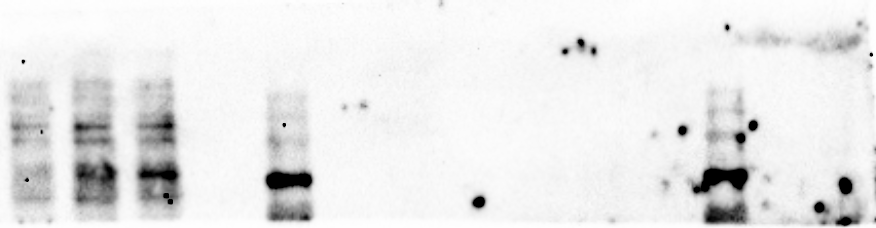

Supplement: Figure 6—figure supplement 1—source data 2. [file elife-85167-fig6-figsupp1-data2.zip › Figure 6-figure supplement 1-source data 2/Originals/V5/originals/220505 V5_44 V5.tif]

Figure 6-figure supplement 1

A

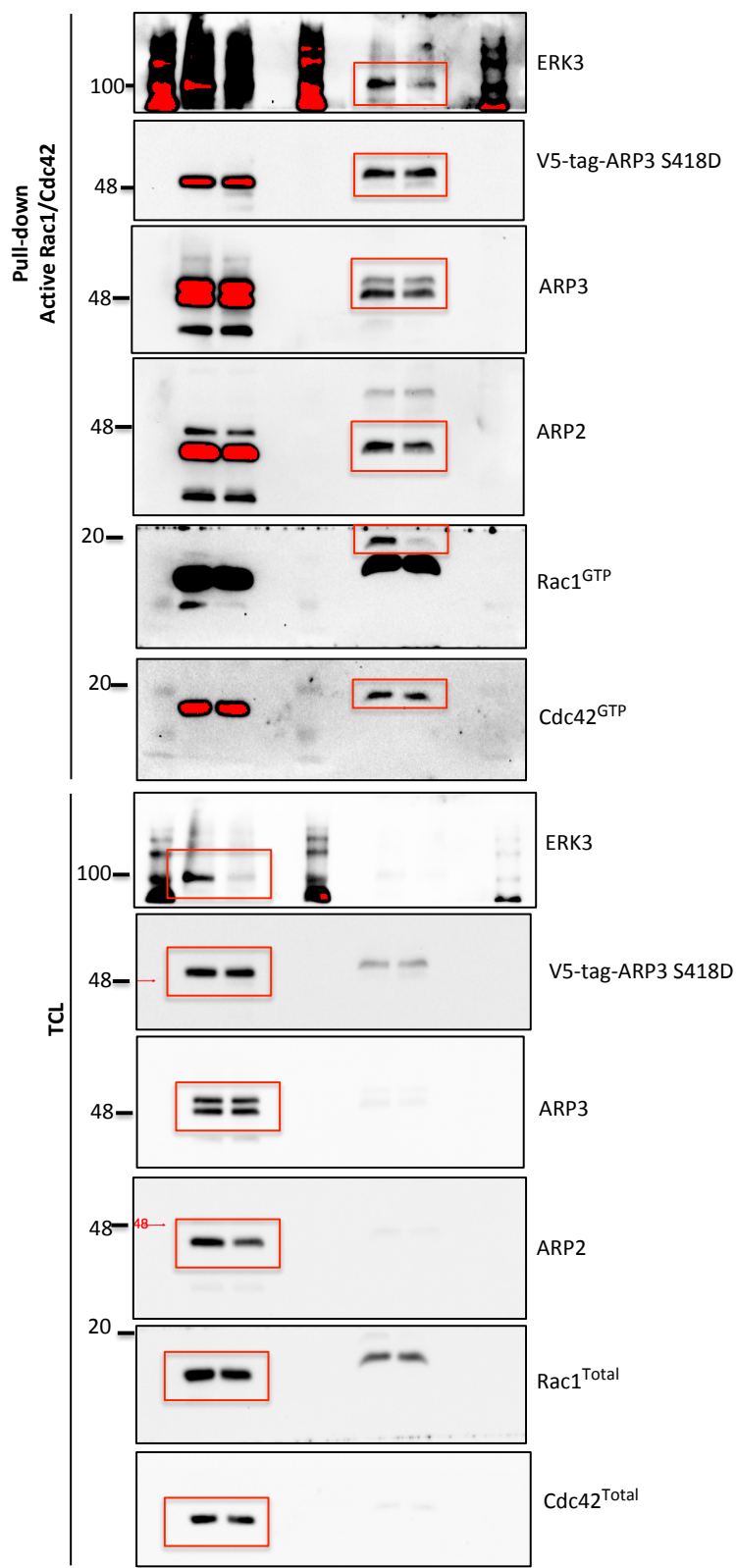

Supplement: Figure 6—figure supplement 2—source data 1. [file elife-85167-fig6-figsupp2-data1.zip › Figure 6-figure supplement 2-source data 1/Figure 6-figure supplement 2A-source data.pdf]

Figure 7

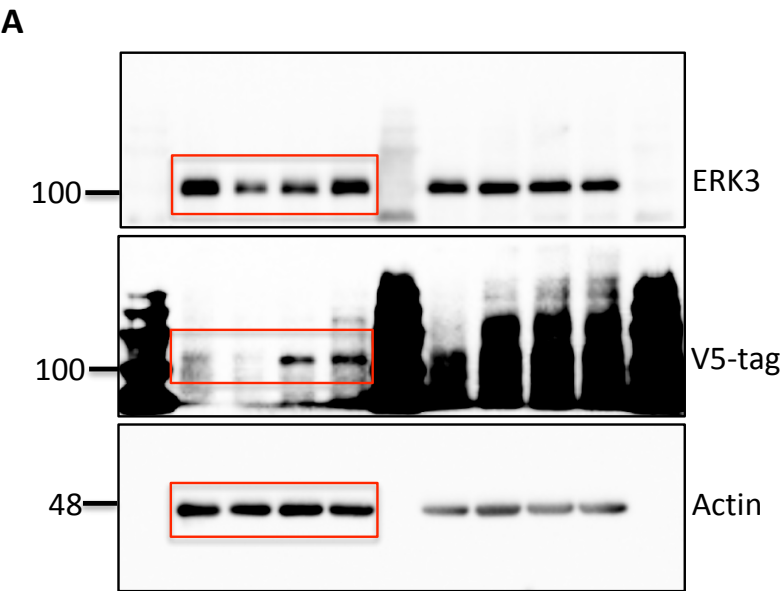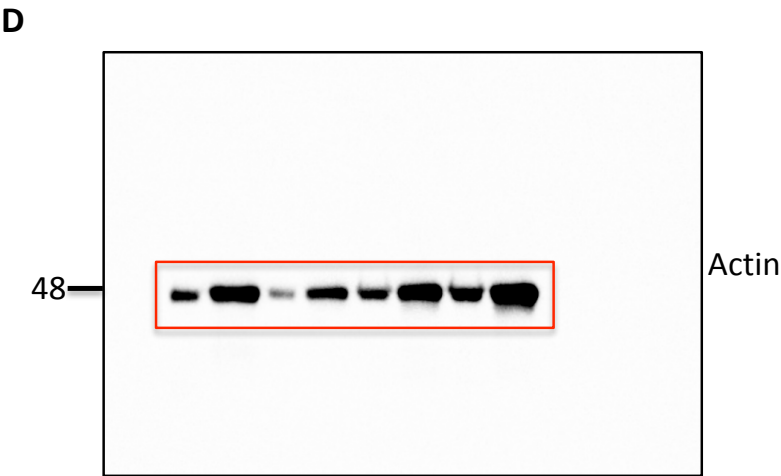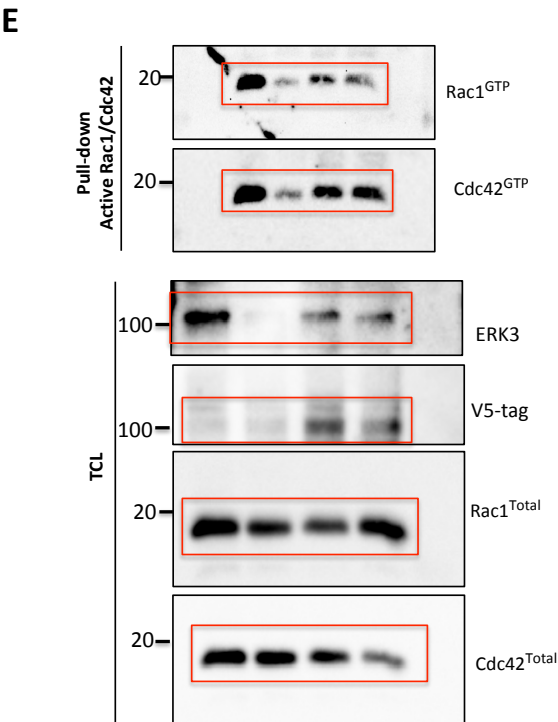

Supplement: Figure 7—source data 1. [file elife-85167-fig7-data1.zip › Figure 7 -source data 1/Figure 7A, 7D and 7E-source data.pdf]

Figure 7-figure supplement 1

A

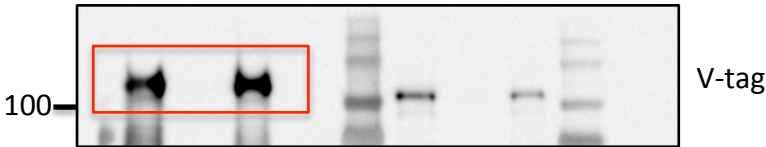

Supplement: Figure 7—source data 1. [file elife-85167-fig7-data1.zip › Figure 7 -source data 1/Figure 7E-source data/Figure 7-figure supplement 1A-source data.pdf]
