# Supplementary material for: ERK3/MAPK6 dictates CDC42/RAC1 activity and ARP2/3-dependent actin polymerization
Source: eLife. 2023 Apr 14;12:e85167. doi: 10.7554/eLife.85167 (PMC10191626; doi:10.7554/eLife.85167)
Supplement: Figure 2—figure supplement 4—source data 1. [file elife-85167-fig2-figsupp4-data1.zip › Figure 2-figure supplement 4-source data/Figure 2-figure supplement 4.pptx]

## Slide 1
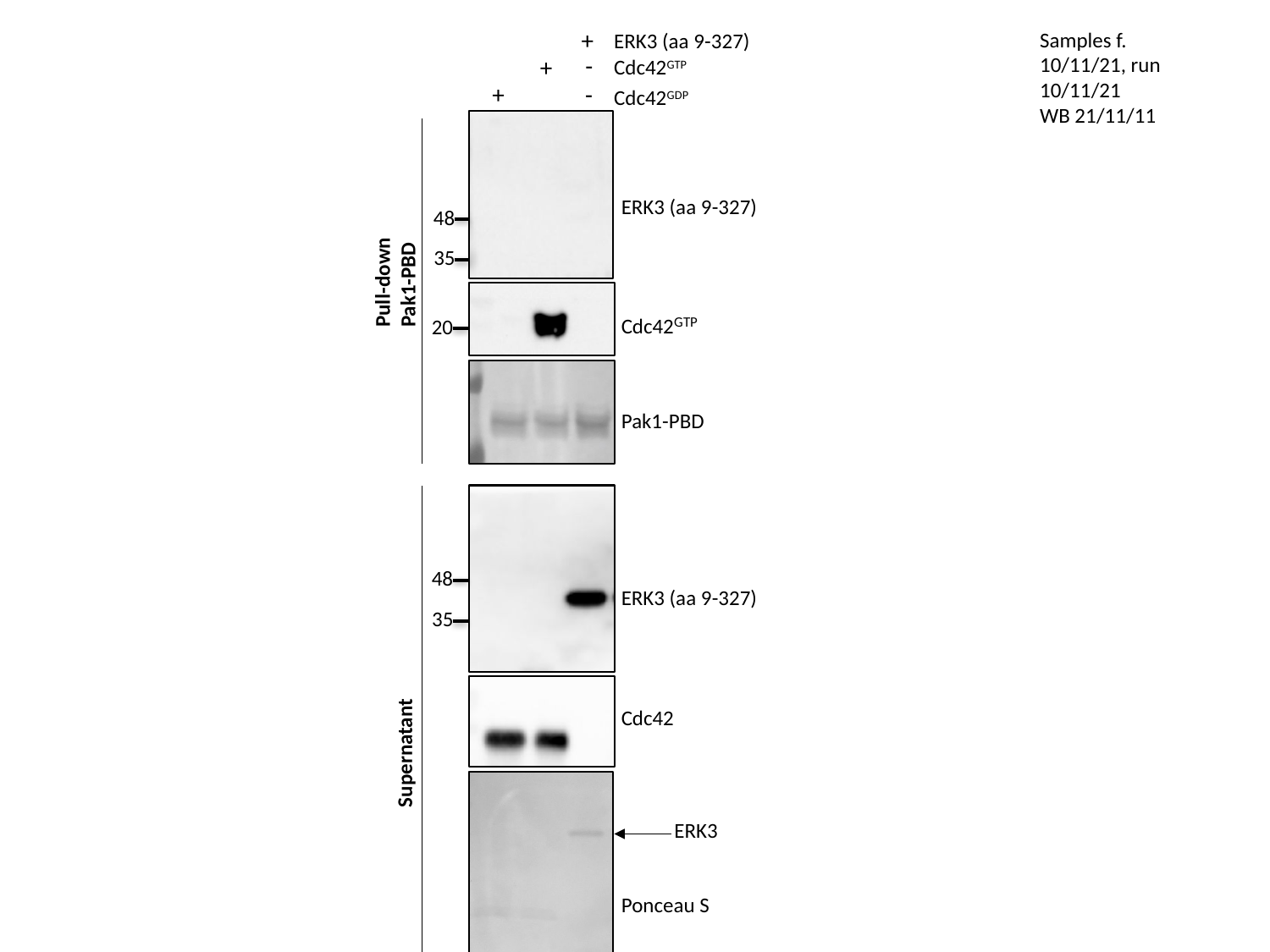

+
Samples f. 10/11/21, run 10/11/21
WB 21/11/11
ERK3 (aa 9-327)
-
+
Cdc42GTP
+
-
Cdc42GDP
ERK3 (aa 9-327)
48
35
Pull-down
Pak1-PBD
Cdc42GTP
20
Pak1-PBD
48
ERK3 (aa 9-327)
35
Cdc42
Supernatant
ERK3
Ponceau S
