# Supplementary material for: ERK3/MAPK6 dictates CDC42/RAC1 activity and ARP2/3-dependent actin polymerization
Source: eLife. 2023 Apr 14;12:e85167. doi: 10.7554/eLife.85167 (PMC10191626; doi:10.7554/eLife.85167)

Figure 3-figure supplement 1

B

In vitro kinase assay
Substrate- MK5

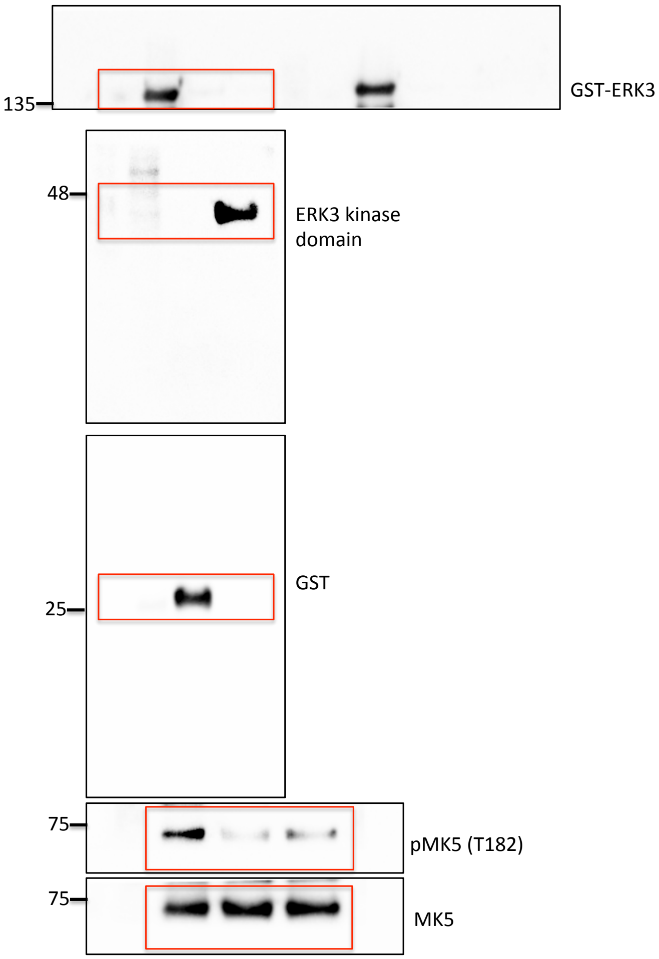

In vitro kinase assay
Substrate- MBP

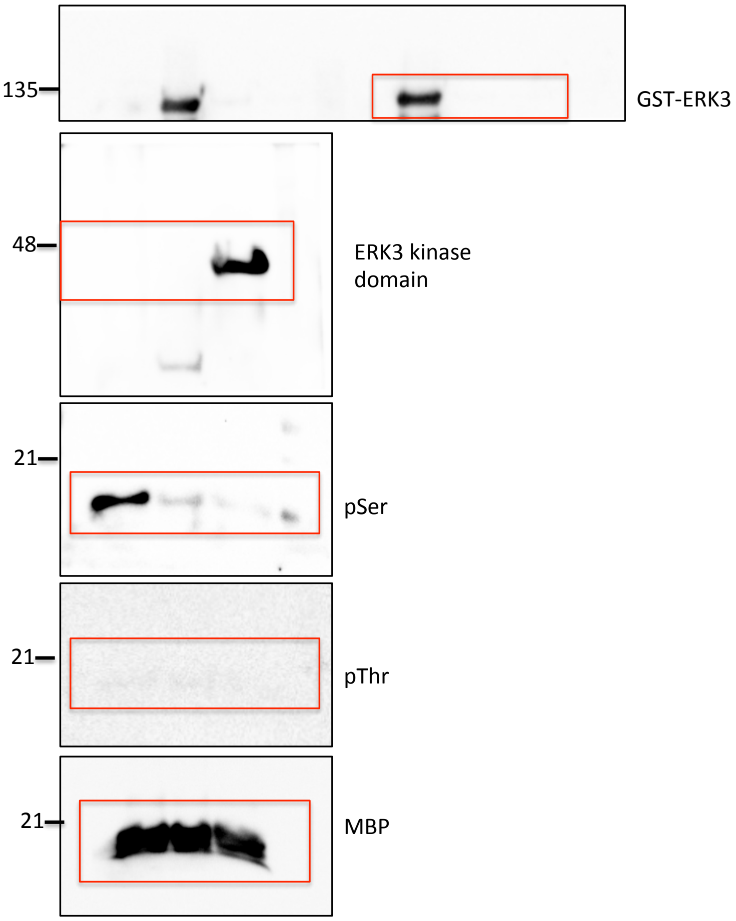

Supplement: Figure 6—figure supplement 1—source data 2. [file elife-85167-fig6-figsupp1-data2.zip › Figure 6-figure supplement 1-source data 2/Figure 6-figure supplement 1B-source data.pdf]
